# Fine-Tuning Mepiquat Chloride Application in Soybean: Interactive Effects of Concentration and Growth Stage on Photosynthetic Apparatus, Carbohydrate Metabolism, and Grain Yield

**DOI:** 10.3390/plants15152381

**Published:** 2026-08-03

**Authors:** Yuanqi Ma, Zhao Li, Yumeng Zhu, Jiaqi Li, Zhipeng Qu, Xinyu Zhou, Jixuan Sun, Wei Zhao, Shoukun Dong

**Affiliations:** 1 Agricultural College, Northeast Agricultural University, Harbin 150030, China; 15663010607@163.com (Y.M.); lizhao_4180@163.com (Z.L.); 15845890950@163.com (Y.Z.); m15045512966_1@163.com (J.L.); zxiny0121@163.com (X.Z.); sunjixuan621@163.com (J.S.); zhaoweiscmy@163.com (W.Z.); 2 Hulunbuir Agricultural Technology Extension Center, Hulunbuir 021008, China; zhipengqu@126.com

**Keywords:** soybean, mepiquat chloride, photosynthetic characteristics, carbohydrates, yield

## Abstract

Soybean is an important protein source, economic crop, and strategic agricultural product, which is related to nutrition and health, industrial development, and food security. Mepiquat chloride (MC) can influence yield, but its effectiveness depends on application timing and concentration. We hypothesized that optimal MC application enhances photosynthetic efficiency and carbohydrate metabolism, thereby increasing yield. A pot experiment was conducted using two cultivars (HN44, HN65) with four MC concentrations (50–400 mg/L) applied at seedling (V3), flowering (R1), or both stages (V3 + R1). Dry matter, photosynthesis, carbohydrates, and yield were evaluated. MC significantly altered dry matter partitioning; 200 mg/L at seedling stage increased total dry matter in HN65 at R6. MC enhanced net photosynthetic rate and PSII efficiency in a concentration-dependent manner, particularly improving photoprotective capacity in HN65. Leaf carbohydrate accumulation was also affected. Yield gains were stage- and cultivar-specific: seedling-stage 200 mg/L increased grain weight per plant in HN65, while flowering-stage 50 mg/L was most effective for HN44. Combined applications (200–400 mg/L) produced the highest yields for both cultivars. This is the first comparative evidence that HN44 and HN65 exhibit divergent yield responses to MC timing—HN44 benefits from flowering-stage, HN65 from seedling-stage application. Optimizing MC concentration and timing enhances yield by modulating photosynthesis and assimilate partitioning in a cultivar-dependent manner.

## 1. Introduction

Soybean (*Glycine max* (L.) Merr.) is a globally significant source of oil and protein; however, China’s domestic production satisfies less than 20% of its demand. This discrepancy underscores an urgent need to enhance yield per unit area through advanced agronomic practices [1,2,3,4,5,6]. The application of plant growth regulators (PGRs) has emerged as a promising strategy for optimizing crop growth and improving productivity [7,8,9]. Among PGRs, mepiquat chloride (MC) is a widely utilized plant growth retardant recognized for its ability to suppress vegetative growth while enhancing reproductive development in various crops, including cotton and soybean [10,11].

MC, characterized by low toxicity and minimal environmental pollution, has been widely evaluated in a range of crops, including sugarcane [12], rice (*Oryza sativa* L.) [13], soybean [11], and sesame [14]. Previous studies have extensively investigated the effects of MC on stem growth characteristics, yield formation, photosynthetic physiology, and other aspects of crop growth and development. Previous studies in cotton have demonstrated that MC effectively suppresses vegetative growth and can substitute for manual topping, with effects becoming more pronounced at higher concentrations [11,15]. These findings suggest that MC is a safe and effective PGR. However, studies on the effects of MC application at different concentrations and developmental stages in soybean remain limited. In particular, the mechanisms by which MC regulates photosynthetic characteristics and yield formation in soybean have not yet been fully elucidated.

In the present study, two soybean cultivars, Heinong 44 (HN44) and Heinong 65 (HN65), were used as experimental materials in a pot experiment. Different concentrations of MC were applied at the seedling stage (V3), flowering stage (R1), and at both stages (V3 + R1) to evaluate their effects on soybean growth and yield and to identify the optimal application stage and concentration for soybean production. This study further examined the responses of photosynthetic characteristics and photosynthate accumulation to MC application under different treatment regimes. The results provide a theoretical basis for the rational use of MC in soybean production in China.

### 1.1. Effects of MC on Photosynthetic Characteristics and Crop Photosynthates

Application of MC can alter stomatal conductance (Gs) and thereby affect plant photosynthesis [16]. In some cases, MC treatment may reduce photosynthetic rate and carbon fixation, thereby influencing carbohydrate metabolism [17]. Ma further showed that under different irrigation regimes, MC application effectively enhanced the photosynthetic capacity, Gs, and Tr of cotton [18]. Wang demonstrated that MC significantly enhanced plant photosynthetic performance, as reflected by significant increases in chlorophyll fluorescence parameters such as ΦII and Fv/Fm [19]. However, the interactive effects of MC concentration and application timing on photosynthetic regulation across various soybean cultivars are still not well understood. The efficiency of carbohydrate transport and partitioning is a key determinant of crop yield [20]. Therefore, a detailed investigation of carbohydrate accumulation and their interrelationships in soybean leaves is essential for understanding the effects of MC on photoassimilate partitioning.

Application of MC may inhibit photosynthesis and thereby limit the production of photoassimilates [21]. Under such conditions, the accumulation of sucrose, hexoses, and starch in leaves may increase, resulting in an imbalance between the production and utilization of photosynthetic products. This imbalance can further restrict photoassimilate synthesis and their translocation to sink tissues [22]. Tang showed that MC application increased sucrose and starch contents per unit leaf area [23]. In cotton, Tung found that MC reduced photosynthesis by promoting the accumulation of sucrose, starch, and hexoses in leaves, thereby decreasing photosynthetic output [24]. However, Shi reported contrasting results, showing that MC treatment reduced the contents of soluble sugars, starch, and sucrose in leaves at 20 d after treatment, but improved actual photosynthetic efficiency at 40 d after treatment and increased biomass accumulation in reproductive organs at 50–60 d after treatment [25]. These contrasting results suggest that the effect of MC on carbohydrate partitioning is complex and may depend on the timing of application and the genotype of the crop, indicating a need for further investigation.

### 1.2. Effects of MC on Dry Matter Accumulation and Crop Yield

MC primarily affects plant growth by altering endogenous hormone levels, particularly through the inhibition of gibberellin biosynthesis. Its regulatory effects on plant growth and development are highly dependent on the timing and concentration of application [26]. Cregan demonstrated that greater dry matter accumulation during the full pod stage resulted in a higher proportion of pod dry matter at maturity [27]. In cowpea, Reddy observed that MC application increased stem dry weight, pod dry weight, and total dry weight [28]. Similarly, Wang reported that under drought stress, MC application in soybean increased leaf and stem dry weight while reducing plant height [21]. Hou further demonstrated, through simulated drought experiments in soybean, that MC application increased dry matter accumulation in both aboveground and belowground plant tissues [29].

Previous studies have reported yield increases of 5–15% with appropriate MC application in soybean and common bean, attributed to increased pod number, seed number, and hundred-seed weight [9,30,31,32], but this response is highly dependent on genotype and application timing, which remains poorly characterized in soybean. The application of MC in modern agricultural production is of considerable importance for crop management [33]. Numerous studies have shown that MC can optimize plant architecture and contribute to the formation of a high-yield plant type [18,19,34]. The establishment of a high-yield plant architecture improves the translocation efficiency of assimilates from vegetative to reproductive organs, thereby enhancing dry matter accumulation and partitioning during crop growth [35], without negatively affecting maturity or yield [36]. Shi compared manual topping, no topping, and MC-based topping in cotton and found that MC-based topping significantly reduced plant height, modified canopy structure, and increased biomass accumulation in reproductive organs, thereby markedly improving yield [37]. Malima investigated the effects of MC application at different cotton growth stages and showed that appropriate MC application increased both yield and fiber quality [38].

## 2. Results

### 2.1. Effects of Different MC Concentrations on Dry Matter Accumulation in Soybean

#### 2.1.1. Effects of MC Application at the Seedling Stage on Dry Matter Accumulation in Soybean

**Figure 1:**
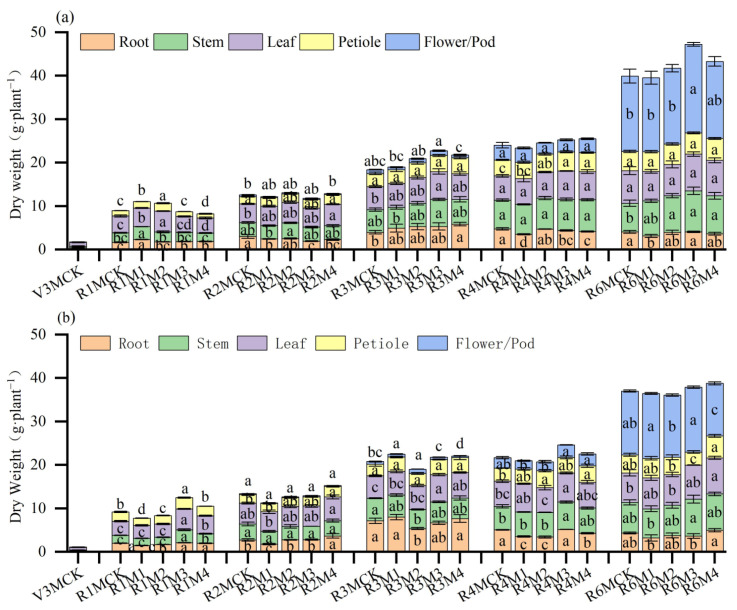
Effects of different concentrations of MC applied at the seedling stage on dry matter accumulation in soybean. Note: (**a**) dry matter accumulation in HN65; (**b**) dry matter accumulation in HN44. Different letters in the figure indicate significant differences at the 0.05 level among treatments on the same date (*p* < 0.05). In the column chart, the treatments represented by each column from left to right are V3 MCK; R1 MCK, R1 M1, R1 M2, R1 M3, R1 M4; R2 MCK, R2 M1, R2 M2, R2 M3, R2 M4; R3 MCK, R3 M1, R3 M2, R3 M3, R3 M4; R4 MCK, R4 M1, R4 M2, R4 M3, R4 M4; R6 MCK, R6 M1, R6 M2, R6 M3, R6 M4.

As shown in Figure 1, foliar application of MC at the seedling stage affected dry matter accumulation in soybean. In HN65, MC application increased total plant dry matter at the R6 stage, with the M3 treatment showing the most pronounced increase. In HN44, total dry matter under M1 and M2 was generally lower than the control (MCK), whereas M3 and M4 resulted in higher dry matter.

#### 2.1.2. Effects of MC Application at the Flowering Stage on Dry Matter Accumulation in Soybean

**Figure 2:**
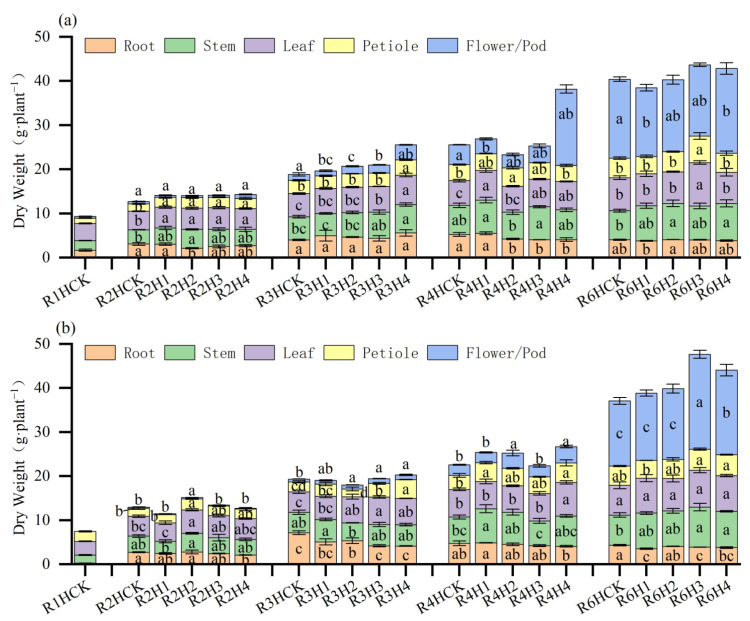
Effects of different concentrations of MC applied at the flowering stage on dry matter accumulation in soybean. Note: (**a**) dry matter accumulation in HN65; (**b**) dry matter accumulation in HN44. Different letters in the figure indicate significant differences at the 0.05 level among treatments on the same date (*p* < 0.05). In the column chart, the treatments represented by each column from left to right are V3 MCK; R1 MCK, R1 M1, R1 M2, R1 M3, R1 M4; R2 MCK, R2 M1, R2 M2, R2 M3, R2 M4; R3 MCK, R3 M1, R3 M2, R3 M3, R3 M4; R4 MCK, R4 M1, R4 M2, R4 M3, R4 M4; R6 MCK, R6 M1, R6 M2, R6 M3, R6 M4.

As shown in Figure 2, foliar application of MC at the flowering stage affected dry matter accumulation in soybean. In HN65, dry matter in MC-treated groups was generally higher than the control after R2, except H3 at R4. In HN44, H4 treatment had higher dry matter than the control across all stages, and at R6, all MC treatments exceeded the control.

#### 2.1.3. Effects of MC Application at Both the Seedling and Flowering Stages on Dry Matter Accumulation in Soybean

**Figure 3:**
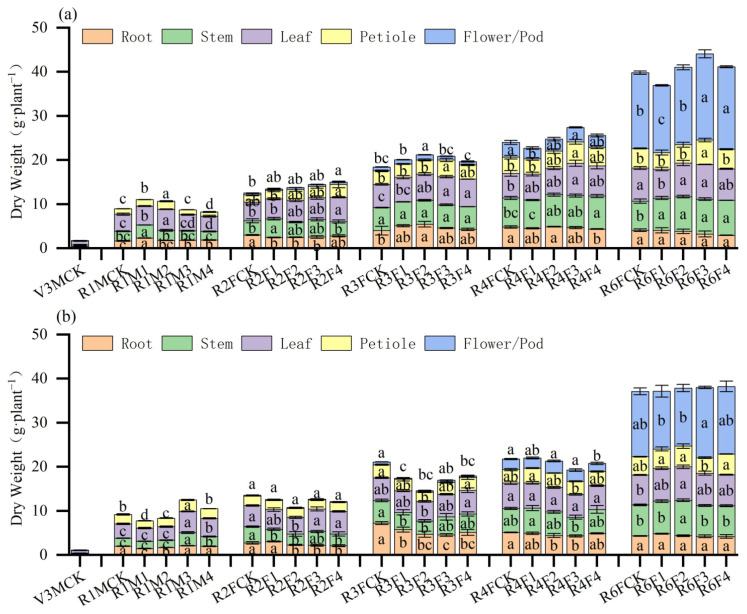
Effects of different concentrations of MC applied at both the seedling and flowering stages on dry matter accumulation in soybean. Note: (**a**) dry matter accumulation in HN65; (**b**) dry matter accumulation in HN44. Different letters in the figure indicate significant differences at the 0.05 level among treatments on the same date (*p* < 0.05). In the column chart, the treatments represented by each column from left to right are V3 MCK; R1 MCK, R1 M1, R1 M2, R1 M3, R1 M4; R2 MCK, R2 M1, R2 M2, R2 M3, R2 M4; R3 MCK, R3 M1, R3 M2, R3 M3, R3 M4; R4 MCK, R4 M1, R4 M2, R4 M3, R4 M4; R6 MCK, R6 M1, R6 M2, R6 M3, R6 M4.

As shown in Figure 3, foliar application of MC at both the seedling and flowering stages affected dry matter accumulation. In HN65, dry matter in all treatment groups was lower than the control at R2 and R3, indicating inhibition, but increased at later stages. In HN44, combined application increased total dry matter at R2 and R3, whereas at R4 and R6, the F1 treatment showed the strongest inhibition.

#### 2.1.4. Interaction Analysis of Application Timing and Sampling Stage on Soybean Dry Matter Accumulation Under Different MC Concentrations

As shown in Table 1, the significance (Sig.) values of the F-tests for the effects of application timing on leaf, stem, petiole, root, and flower/pod dry matter accumulation in HN65 were all greater than 0.05. In addition, the Sig. values for the effects of MC concentration on root dry matter accumulation in HN65 and stem dry matter accumulation in HN44 were also greater than 0.05. For the interaction between application timing and MC concentration, the Sig. values for leaf and stem dry matter accumulation in HN65 were greater than 0.05 as well. These results indicate that MC concentration and the interaction between application timing and concentration had no significant effects on the above-mentioned traits, whereas significant effects were observed for the remaining indicators.

### 2.2. Effects of Different MC Concentrations on Photosynthetic Characteristics in Soybean

#### 2.2.1. Effects of Different MC Concentrations on Photosynthetic Parameters in Soybean

##### Effects of Different MC Concentrations on Pn in Soybean

**Figure 4:**
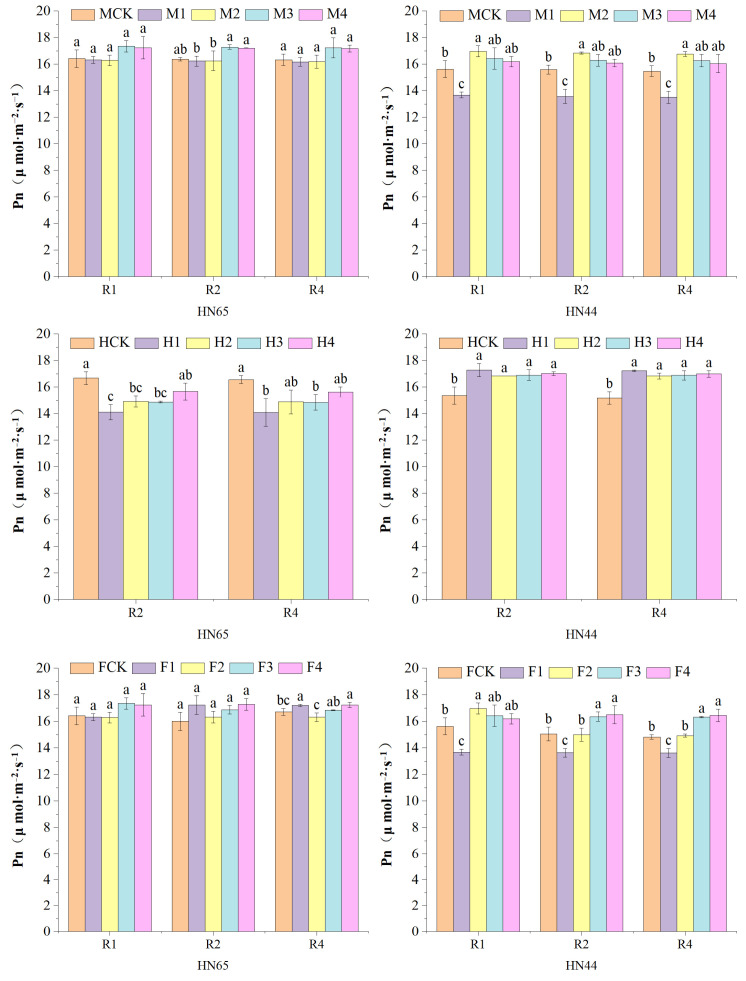
Effects of different concentrations of MC on the net photosynthetic rate of soybean. Note: Different letters in the figure indicate significant difference at the 0.05 level among treatments on the same date (*p* < 0.05).

As shown in Figure 4, seedling-stage MC application increased Pn in HN65 under M3/M4 and in HN44 under M2/M3/M4, while M1 decreased Pn in both cultivars. Flowering-stage MC decreased Pn in HN65 but increased it in HN44. Combined application increased Pn in HN65 at R2 and in HN44 under F3/F4 at R2. In HN65, seedling-stage Pn peaked under M3. Flowering-stage Pn was highest under H4 and lowest under H1. Combined application had the lowest Pn under F3. In HN44, seedling-stage Pn peaked under M2. Flowering-stage Pn was lowest under H2. Combined application gradually increased Pn with concentration.

##### Effects of Different MC Concentrations on Gs in Soybean

**Figure 5:**
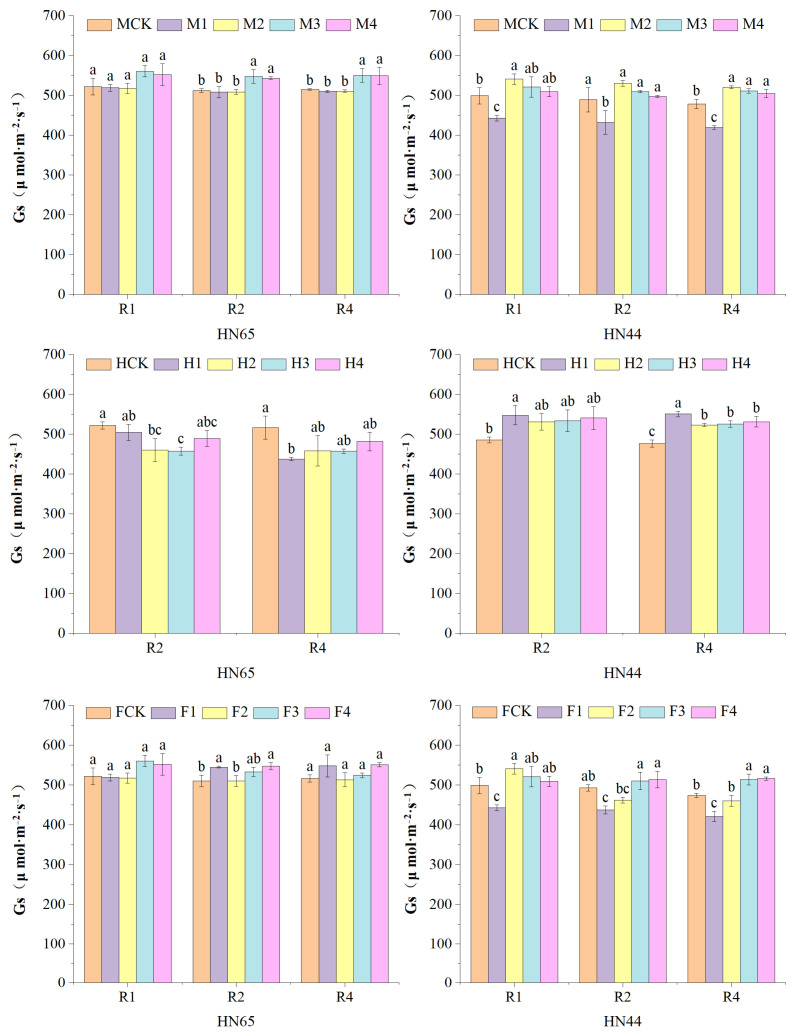
Effects of different concentrations of MC on stomatal conductance in soybean. Note: Different letters in the figure indicate significant difference at the 0.05 level among treatments on the same date (*p* < 0.05).

As shown in Figure 5, seedling-stage MC application increased Gs in both cultivars at R2 and R4. Flowering-stage MC decreased Gs in HN65 but increased it in HN44. Combined application increased Gs in HN65 at R2 and in HN44 under F3/F4 at R2. In HN65, seedling-stage Gs peaked under M3. Flowering-stage Gs was lowest under H3. Combined application had the lowest Gs under F2. In HN44, seedling-stage Gs peaked under M2. Flowering-stage Gs was lowest under H2. Combined application led to a gradual increase in Gs with concentration.

##### Effects of Different MC Concentrations on Tr in Soybean

**Figure 6:**
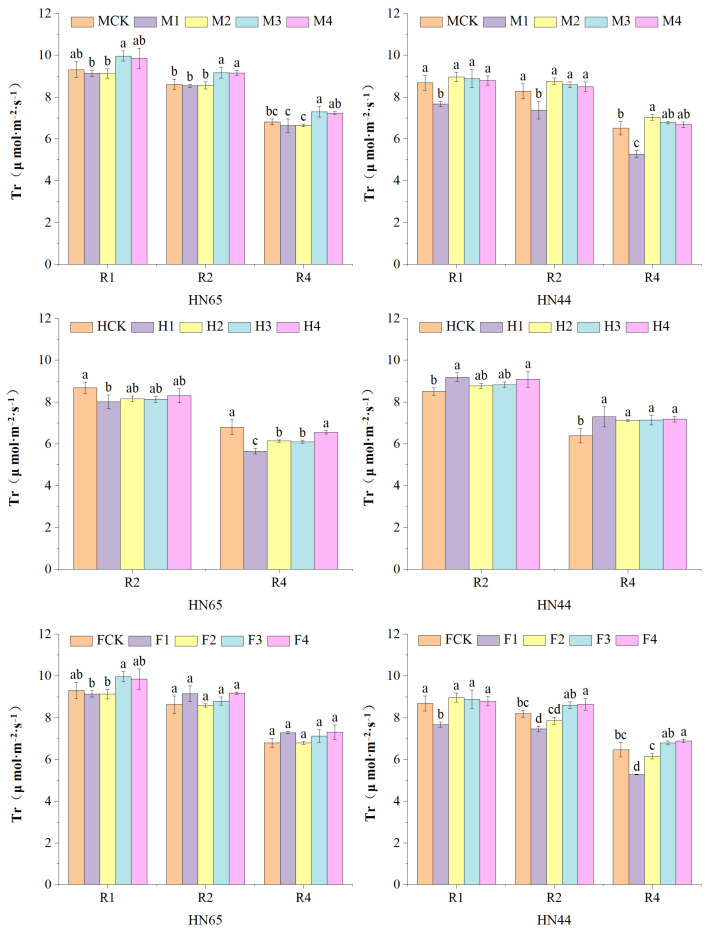
Effects of different concentrations of MC on transpiration rate in soybean. Note: Different letters in the figure indicate significant difference at the 0.05 level among treatments on the same date (*p* < 0.05).

As shown in Figure 6, seedling-stage MC increased Tr in HN65 under M3/M4 and in HN44 under M2/M3/M4, while M1 reduced Tr in both. Flowering-stage MC decreased Tr in HN65 but increased it in HN44. Combined application increased Tr in HN65 under F1/F3/F4 at R2 and in HN44 under F3/F4 at R2. In HN65, seedling-stage Tr peaked under M3. Flowering-stage Tr was highest under H4 and lowest under H1. Combined application had the lowest Tr under F3. In HN44, seedling-stage Tr peaked under M2. Flowering-stage Tr was lowest under H2. Combined application led to a gradual increase in Tr with concentration.

##### Effects of Different MC Concentrations on Ci in Soybean

**Figure 7:**
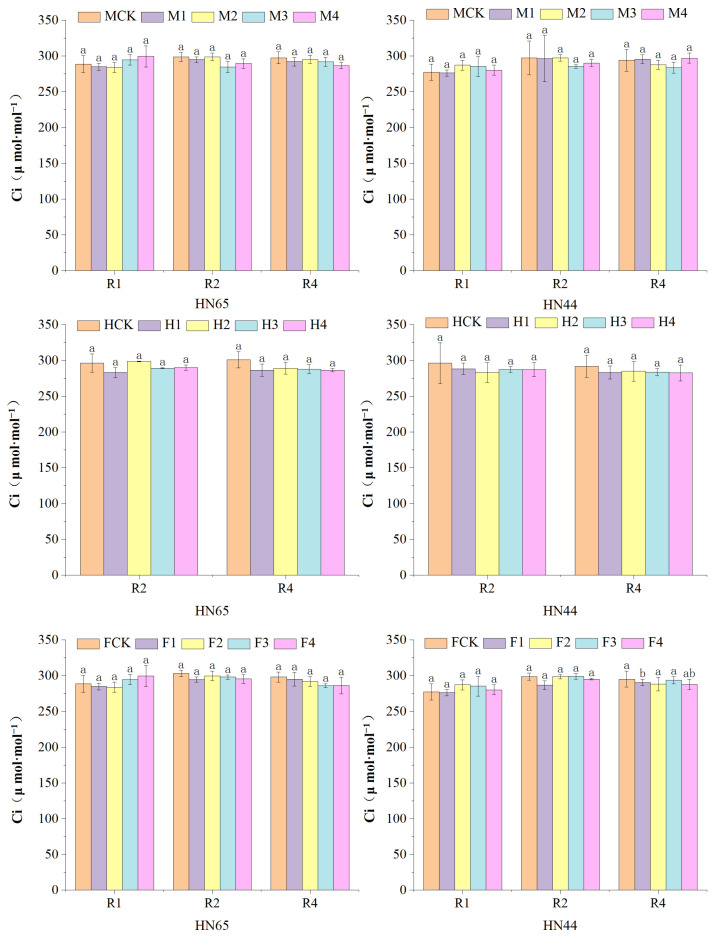
Effects of different concentrations of MC on intercellular CO_2_ concentration in soybean. Note: Different letters in the figure indicate significant difference at the 0.05 level among treatments on the same date (*p* < 0.05).

As shown in Figure 7, MC application at the seedling stage had no significant effect on the Ci of HN65, whereas the combined application at the seedling and flowering stages significantly affected Ci in HN44 at the R4 stage. In general, Ci in both cultivars remained relatively stable at approximately 290 μmol mol^−1^, with no marked fluctuations among treatments.

#### 2.2.2. Effects of Different MC Concentrations on Chlorophyll Fluorescence Parameters in Soybean

##### Effects of Different MC Concentrations on Fv/Fm in Soybean

**Figure 8:**
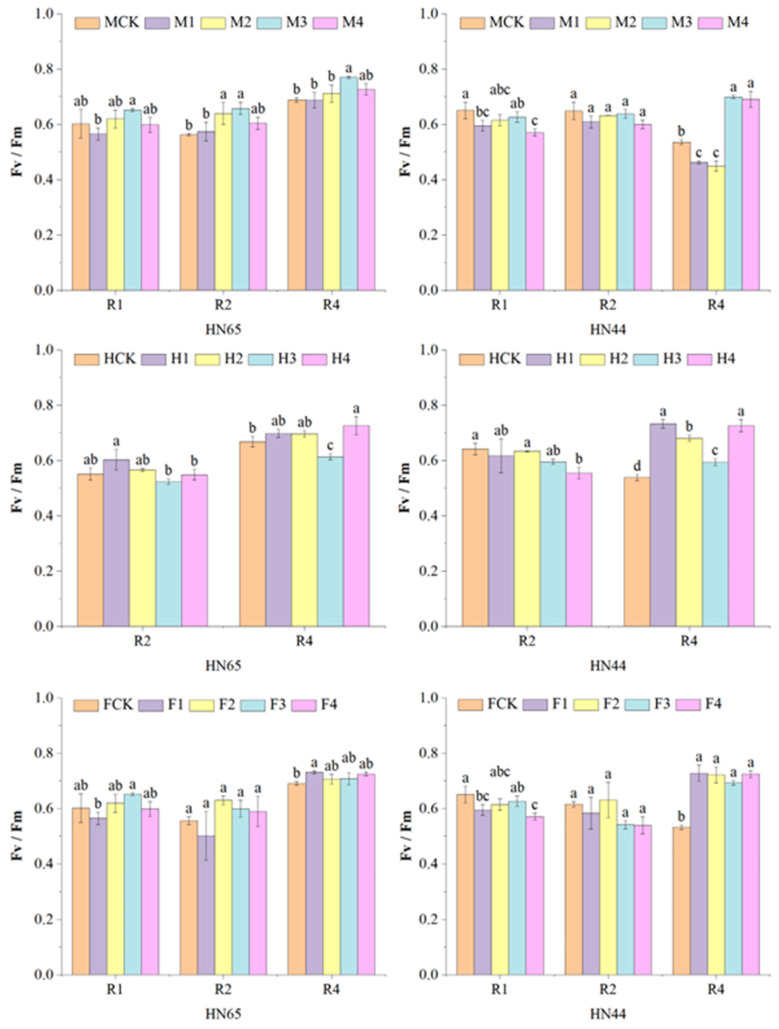
Effects of different concentrations of MC on Fv/Fm in soybean. Note: Different letters in the figure indicate significant difference at the 0.05 level among treatments on the same date (*p* < 0.05).

As shown in Figure 8, MC application reduced Fv/Fm in some treatments of both cultivars, but several treatments showed subsequent recovery. In HN65, seedling-stage Fv/Fm first increased and then decreased with concentration, peaking under M3. Flowering-stage Fv/Fm first decreased and then increased, with the lowest under H3. Combined application led to a similar pattern at R4. In HN44, seedling-stage Fv/Fm peaked under M3. Flowering-stage Fv/Fm at R2 peaked under H2, while at R4, it first decreased and then increased, with the lowest under H3. Combined application at R4 resulted in increases relative to the control.

##### Effects of Different MC Concentrations on qL in Soybean

**Figure 9:**
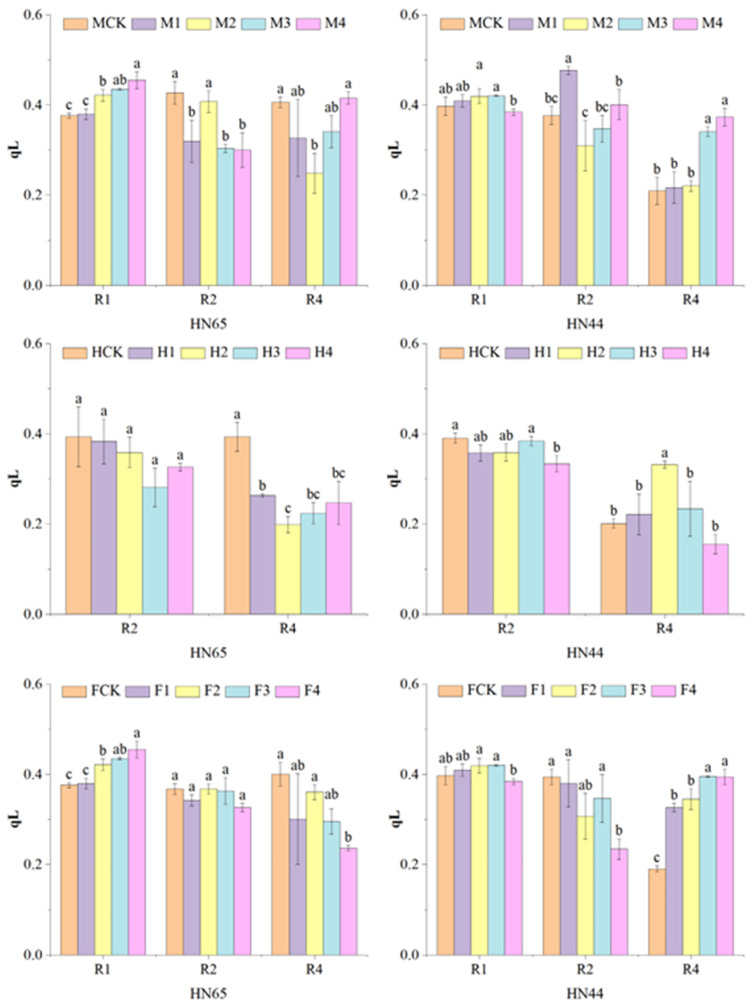
Effects of different concentrations of MC on qL in soybean. Note: Different letters in the figure indicate significant difference at the 0.05 level among treatments on the same date (*p* < 0.05).

As shown in Figure 9, seedling-stage application increased qL in both cultivars, whereas flowering-stage and combined applications generally reduced qL. In HN65, seedling-stage MC application significantly affected qL at R1, R2, and R4. With increasing concentration, qL shifted from a gradual increase to first decreasing and then increasing. Flowering-stage application significantly affected qL at R4, where qL first decreased and then increased, with the lowest under H2. Combined application affected qL at R4, where qL first increased and then decreased, peaking under F2. In HN44, seedling-stage MC application significantly affected qL at R1, R2, and R4. With increasing concentration, qL shifted from first increasing and then decreasing to a gradual increase. Flowering-stage application significantly affected qL at R2 and R4; qL first increased and then decreased, with peaks under H3 (R2) and H2 (R4). Combined application significantly affected qL at R2 and R4; at R4, qL first increased and then decreased. In summary, seedling-stage MC increased qL, whereas flowering-stage and combined applications generally reduced qL, indicating that the effects of MC on photochemical quenching were strongly dependent on application stage.

##### Effects of Different MC Concentrations on NPQ in Soybean

**Figure 10:**
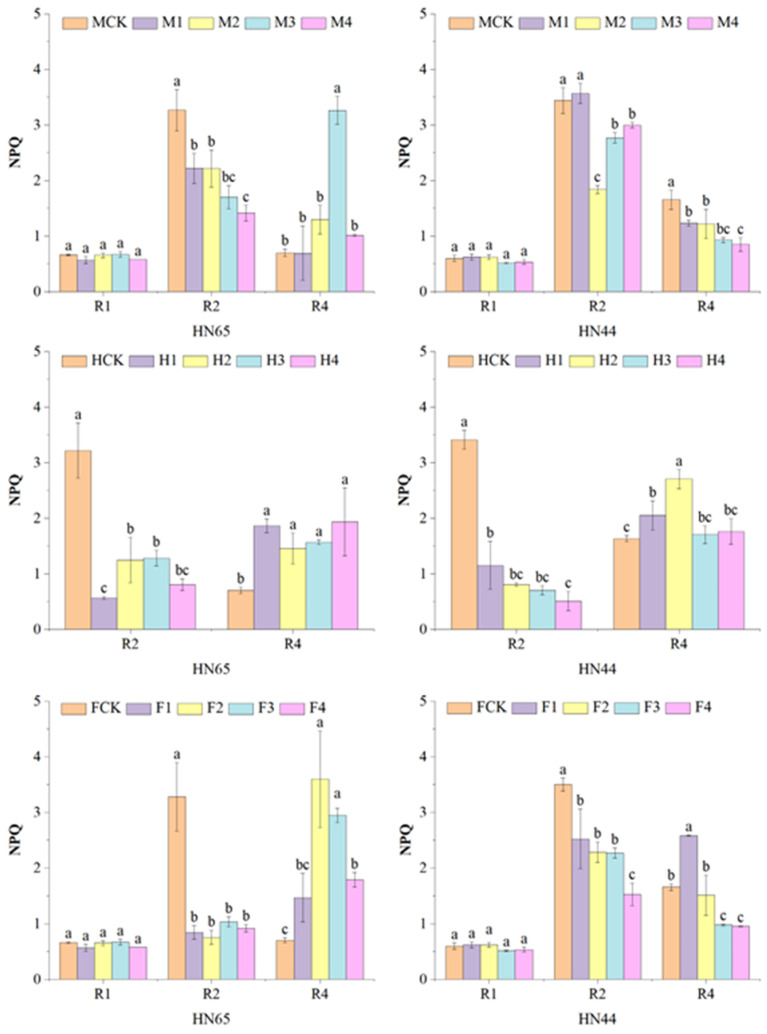
Effects of different concentrations of MC on NPQ in soybean. Note: Different letters in the figure indicate significant difference at the 0.05 level among treatments on the same date (*p* < 0.05).

As shown in Figure 10, seedling-stage MC reduced NPQ in some treatments. At R2, all MC applications generally decreased NPQ, but later, several treatments exceeded the control. In HN65, seedling-stage NPQ at R2 decreased gradually with concentration. Flowering-stage and combined applications increased NPQ at R4. In HN44, seedling-stage NPQ at R2 first increased and then decreased (lowest under M2), while at R4, it decreased gradually. Flowering-stage NPQ at R2 decreased gradually; at R4, NPQ increased relative to the control. Combined application led to a gradual decreasing trend in NPQ.

##### Effects of Different MC Concentrations on ΦII in Soybean

**Figure 11:**
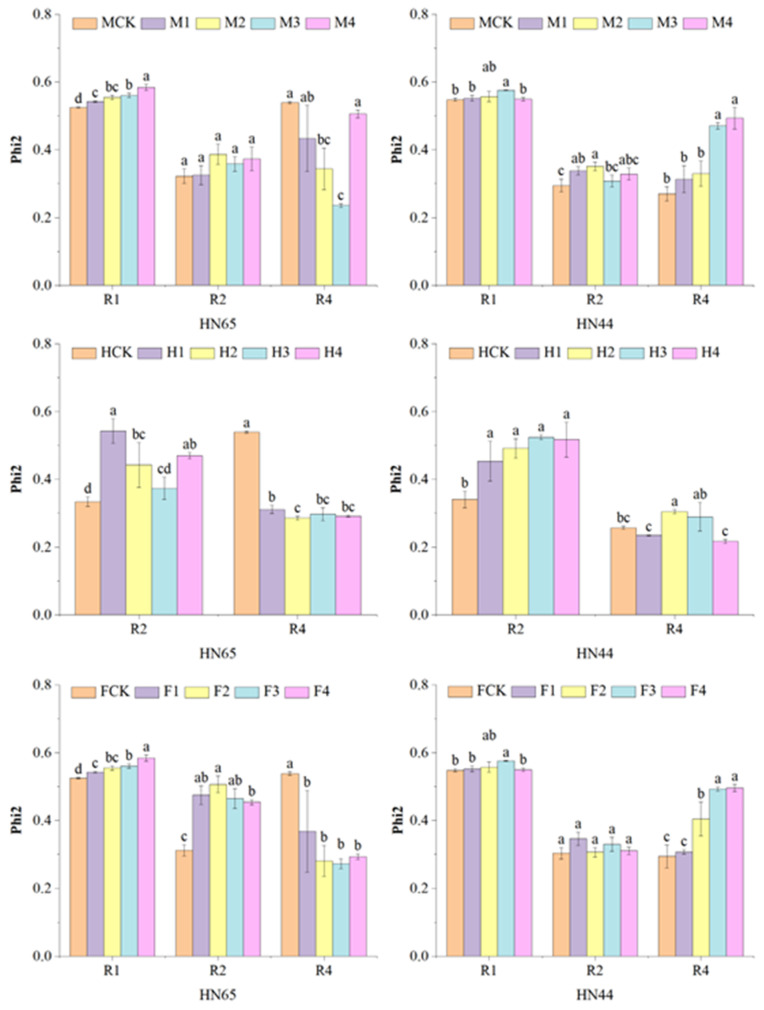
Effects of different concentrations of MC on ΦII in soybean. Note: Different letters in the figure indicate significant difference at the 0.05 level among treatments on the same date (*p* < 0.05).

As shown in Figure 11, seedling-stage MC increased ΦII in both cultivars at R1 and R2, whereas flowering-stage MC decreased ΦII in some treatments at R4. In HN65, seedling-stage ΦII shifted from a gradual increase to first decreasing and then increasing. Flowering-stage ΦII at R2 first decreased and then increased, with the lowest under H3. Combined application increased ΦII at R2; at R4, ΦII first decreased and then increased, lowest under F3. In HN44, seedling-stage ΦII first increased and then decreased at R2, but increased gradually at R4. Flowering-stage ΦII first increased and then decreased, with peaks under H3 (R2) and H2 (R4). Combined application at R4 increased ΦII gradually.

##### Effects of Different MC Concentrations on ΦNO in Soybean

**Figure 12:**
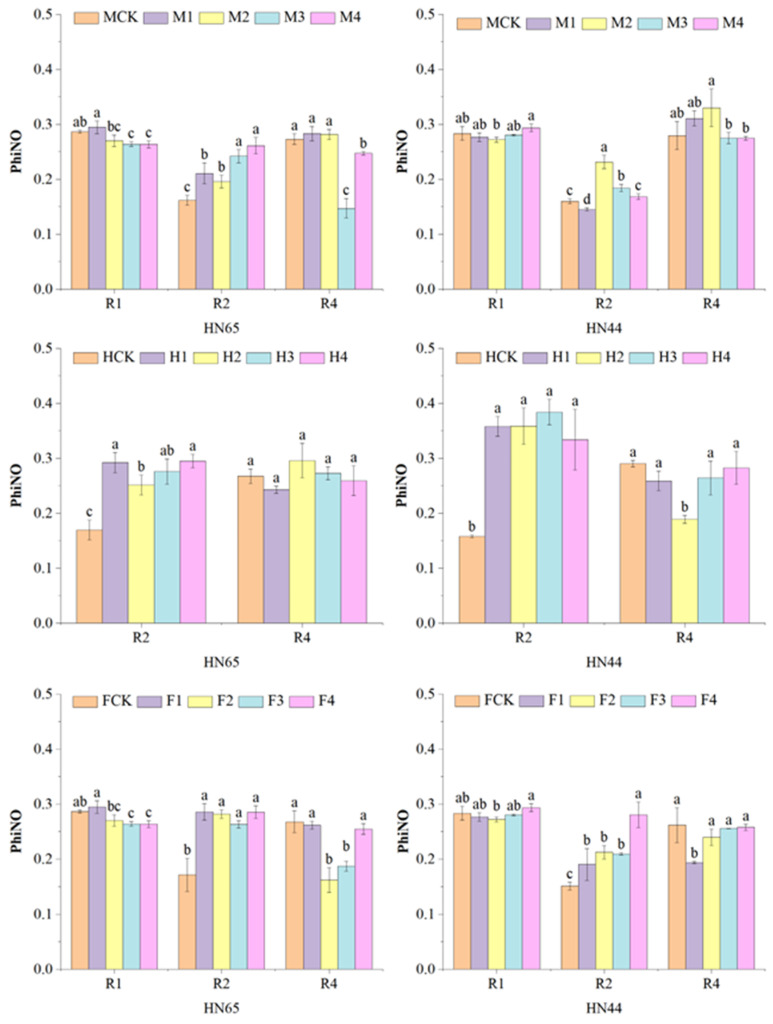
Effects of different concentrations of MC on ΦNO in soybean. Note: Different letters in the figure indicate significant difference at the 0.05 level among treatments on the same date (*p* < 0.05).

As shown in Figure 12, seedling-stage MC reduced ΦNO under M3/M4 at R4. Flowering-stage MC reduced ΦNO in all HN44 treatments and in H1/H4 of HN65 at R4. Combined application increased ΦNO at R2 but decreased it at R4. In HN65, seedling-stage ΦNO at R2 increased relative to the control. Flowering-stage ΦNO at R2 first decreased and then increased, with the lowest under H2. Combined application increased ΦNO at R2. In HN44, seedling-stage ΦNO at R2 changed variably among treatments. Flowering-stage ΦNO at R2 first increased and then decreased (highest under H3), while at R4, it first decreased and then increased (lowest under H2). Combined application led to a gradual increasing trend in ΦNO at R4.

##### Effects of Different MC Concentrations on ΦNPQ in Soybean

**Figure 13:**
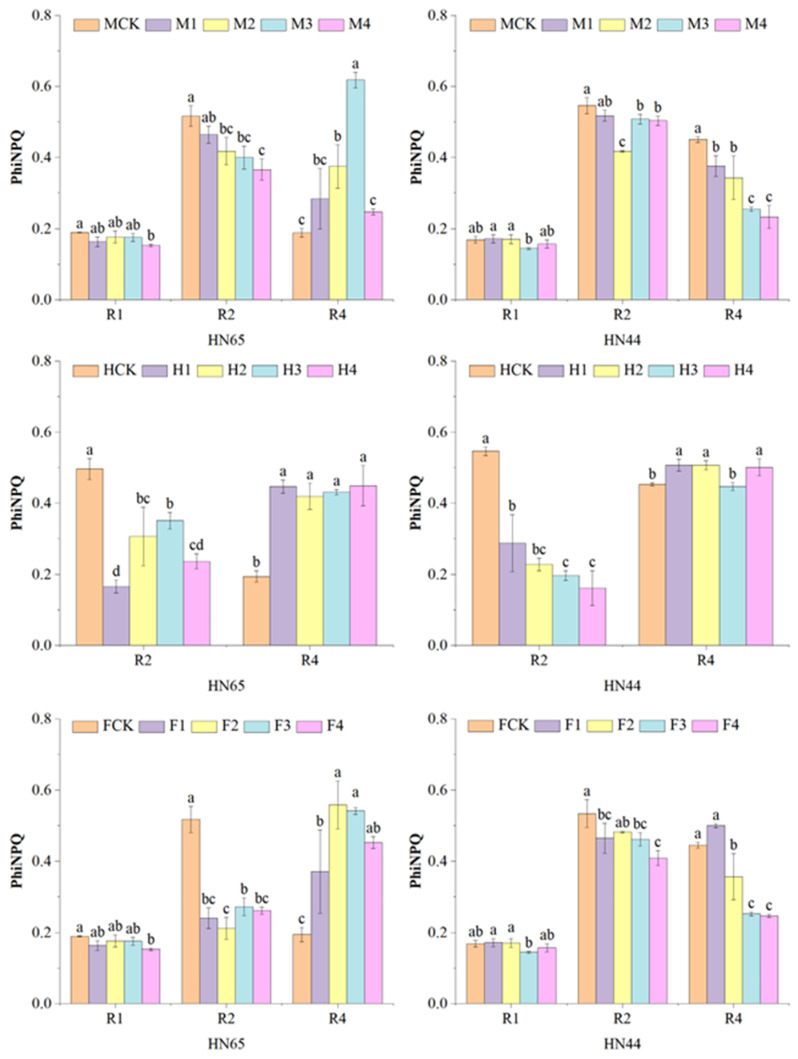
Effects of different concentrations of MC on ΦNPQ in soybean. Note: Different letters in the figure indicate significant difference at the 0.05 level among treatments on the same date (*p* < 0.05).

As shown in Figure 13, MC reduced ΦNPQ at R2, but at R4, it exceeded the control in all HN65 treatments and in H1/H2/H4 of HN44. In HN65, seedling-stage, flowering-stage, and combined applications all increased ΦNPQ at R4 relative to the control. In HN44, seedling-stage ΦNPQ at R1 showed minor changes. Flowering-stage ΦNPQ at R2 decreased gradually, while at R4, it first decreased and then increased (lowest under H3). Combined application led to a gradual decreasing trend in ΦNPQ at R4.

#### 2.2.3. Interaction Analysis of Application Timing and Sampling Stage on Photosynthesis-Related Parameters in Soybean Under Different MC Concentrations

As shown in Table 2, the significance (Sig.) values of the F-tests for the effects of application timing on intercellular Ci and ΦII in HN65, as well as on Ci and ΦII in HN44, were all greater than 0.05. The Sig. values for the effects of MC concentration on Ci in HN65 and on Ci and Fv/Fm in HN44 were also greater than 0.05. For the interaction between application timing and MC concentration, the Sig. values for Ci and ΦII in HN65 and for Ci in HN44 were all greater than 0.05. In addition, for the interaction between the sampling stage and MC concentration, the Sig. values for Pn, Gs, Tr, and Fv/Fm in HN65, as well as for Pn, Gs, Tr, and Ci in HN44, were all greater than 0.05. These results indicate that application timing, MC concentration, the interaction between application timing and concentration, and the interaction between sampling stage and concentration had no significant effects on the above-mentioned traits, whereas significant effects were detected for the remaining parameters.

### 2.3. Effects of Different MC Concentrations on Main Carbohydrate Contents in Soybean

#### 2.3.1. Effects of Different MC Concentrations on Starch Content in Soybean

##### Effects of MC Application at the Seedling Stage on Starch Content in Soybean

As shown in Table 3, seedling-stage application of MC significantly affected starch content in soybean leaves, increasing starch content in all treatments of HN65 at the R1 and R4 stages and in all treatments of HN44 at the R3 and R4 stages. In HN65, seedling-stage MC application significantly affected leaf starch content at the R1, R3, R4, and R6 stages. With increasing MC concentration, starch content in the treatment groups first increased and then decreased at the R1 and R2 stages, whereas at the R3, R4, and R6 stages, it first decreased and then increased. In HN44, seedling-stage MC application significantly affected starch content at the R1, R2, R3, R4, and R6 stages. With increasing MC concentration, starch content in the treatment groups first increased and then decreased at the R1, R2, R3, and R4 stages, whereas at the R6 stage, it first decreased and then increased. Over time, starch content under the M3 treatment showed a pattern of first increasing and then decreasing, with the highest value observed at the R2 stage.

##### Effects of MC Application at the Flowering Stage on Starch Content in Soybean

As shown in Table 4, flowering-stage application of MC significantly affected starch content in soybean leaves. In HN65, MC treatment increased starch content in all treatments at the R4 stage. Over time, starch content under the H2 treatment first decreased and then increased, whereas under the H3 treatment, it decreased gradually. In HN44, starch content under the H2 treatment also showed a pattern of first decreasing and then increasing over time, whereas the H1 and H4 treatments showed a gradual decline. MC application increased starch content in all treatments of HN44 at the R3 and R4 stages. In HN65, flowering-stage MC application significantly affected leaf starch content at the R2, R3, R4, and R6 stages. With increasing MC concentration, starch content in the treatment groups first increased and then decreased at the R2 and R3 stages, whereas at the R4 and R6 stages, it first decreased and then increased. In HN44, flowering-stage MC application significantly affected leaf starch content at the R2, R3, R4, and R6 stages. With increasing MC concentration, leaf starch content in the treatment groups first decreased and then increased at the R2, R3, and R4 stages, with the lowest values observed under the H4, H3, and H2 treatments, respectively.

##### Effects of MC Application at Both the Seedling and Flowering Stages on Starch Content in Soybean

As shown in Table 5, the combined application of MC at the seedling and flowering stages significantly affected leaf starch content in soybean. Over time, starch content in HN65 under the F1 and F2 treatments first increased and then decreased, with the highest value observed at the R2 stage. In HN44, starch content under the F1 and F4 treatments first decreased and then increased over time, and the highest value was also observed at the R2 stage. In HN65, combined application at the seedling and flowering stages significantly affected leaf starch content at the R2, R3, R4, and R6 stages. With increasing MC concentration, starch content in the treatment groups showed a gradual decrease at R2, a pattern of first increasing and then decreasing at R3, and a pattern of first decreasing and then increasing at R6. In HN44, combined application at the seedling and flowering stages significantly affected leaf starch content at the R2, R3, R4, and R6 stages. With increasing MC concentration, leaf starch content increased gradually at R2, whereas at R4 and R6, it first increased and then decreased, with the highest values observed under the F2 and F3 treatments, respectively.

#### 2.3.2. Effects of Different MC Concentrations on Soluble Sugar Content in Soybean

##### Effects of MC Application at the Seedling Stage on Soluble Sugar Content in Soybean

As shown in Table 6, soluble sugar content in HN65 under all treatments showed a pattern of first increasing and then decreasing over time. The highest soluble sugar content was observed at the R3 stage under the M1 and M3 treatments, whereas under the M2 and M4 treatments, the highest values occurred at the R4 stage. In HN44, soluble sugar content under the M1, M2, and M3 treatments also showed a trend of first increasing and then decreasing over time. The highest values occurred at the R3 stage under the M1 and M3 treatments and at the R4 stage under the M2 treatment. Seedling-stage MC application significantly affected leaf soluble sugar content in HN65 at the R1, R2, R3, R4, and R6 stages. With increasing MC concentration, soluble sugar content in the treatment groups decreased gradually at the R1 and R2 stages, whereas at the R3, R4, and R6 stages, it first decreased and then increased, with the lowest values observed under the M2, M3, and M4 treatments, respectively. Seedling-stage MC application also significantly affected leaf soluble sugar content in HN44 at the R1, R2, R3, R4, and R6 stages. With increasing MC concentration, soluble sugar content in the treatment groups increased gradually at the R1 stage, whereas at the R2, R3, R4, and R6 stages, it first increased and then decreased, with the highest values all observed under the M3 treatment.

##### Effects of MC Application at the Flowering Stage on Soluble Sugar Content in Soybean

As shown in Table 7, soluble sugar content in HN65 under the HCK treatment showed a pattern of first increasing and then decreasing over time, with the highest value observed at the R3 stage. In HN44, soluble sugar content under all treatments also showed a trend of first increasing and then decreasing over time. The highest values occurred at the R3 stage under the HCK and H1 treatments, whereas under the H2, H3, and H4 treatments, the highest values were observed at the R4 stage. Flowering-stage MC application significantly affected leaf soluble sugar content in HN65 at the R2, R3, R4, and R6 stages. With increasing MC concentration, soluble sugar content in the treatment groups first decreased and then increased at the R2 and R3 stages, whereas at the R4 and R6 stages, it first increased and then decreased. At the R2, R4, and R6 stages, leaf soluble sugar content in all treatment groups was lower than that in HCK. Flowering-stage MC application also significantly affected leaf soluble sugar content in HN44 at the R2, R3, R4, and R6 stages. With increasing MC concentration, soluble sugar content in the treatment groups increased gradually at the R2 stage, whereas at the R3, R4, and R6 stages, it first increased and then decreased, with the highest values observed under the H3, H2, and H1 treatments, respectively.

##### Effects of MC Application at Both the Seedling and Flowering Stages on Soluble Sugar Content in Soybean

As shown in Table 8, the combined application of MC at the seedling and flowering stages reduced soluble sugar content in all treatments of HN65 at the R2, R3, and R4 stages. Compared with the corresponding values at the R1 stage, soluble sugar content decreased markedly after the second MC application at the flowering stage. In HN44, soluble sugar content under the FCK treatment showed a pattern of first increasing and then decreasing over time. From R2 to R6, soluble sugar content under the F2, F3, and F4 treatments first decreased and then increased. Combined application at the seedling and flowering stages significantly affected leaf soluble sugar content in HN65 at the R2, R3, R4, and R6 stages. With increasing MC concentration, soluble sugar content in the treatment groups increased gradually at R2, whereas at R4 and R6, it first decreased and then increased, with the highest values observed under the F3 and F2 treatments, respectively. Combined application at the seedling and flowering stages also significantly affected leaf soluble sugar content in HN44 at the R2, R3, R4, and R6 stages. With increasing MC concentration, soluble sugar content in the treatment groups first increased and then decreased at R2, R3, R4, and R6, with the highest values observed under the F3, F2, F2, and F2 treatments, respectively.

#### 2.3.3. Effects of Different MC Concentrations on Sucrose Content in Soybean

##### Effects of MC Application at the Seedling Stage on Sucrose Content in Soybean

As shown in Table 9, sucrose content in HN65 under all treatments showed a pattern of first increasing and then decreasing over time. The highest sucrose content was observed at the R2 stage under the MCK and M3 treatments, at the R3 stage under the M1 and M2 treatments, and at the R4 stage under the M4 treatment. In HN44, sucrose content under all treatments also showed a trend of first increasing and then decreasing over time. The highest values occurred at the R3 stage under the MCK and M3 treatments, at the R2 stage under the M1 and M2 treatments, and at the R4 stage under the M4 treatment. Seedling-stage MC application significantly affected leaf sucrose content in HN65 at the R1, R2, R3, R4, and R6 stages. With increasing MC concentration, sucrose content in the treatment groups first increased and then decreased at the R1, R2, and R3 stages, whereas at the R4 and R6 stages, it first decreased and then increased. Seedling-stage MC application also significantly affected leaf sucrose content in HN44 at the R1, R2, R3, R4, and R6 stages.

##### Effects of MC Application at the Flowering Stage on Sucrose Content in Soybean

As shown in Table 10, sucrose content in HN65 under all flowering-stage treatments showed a pattern of first increasing and then decreasing over time. The highest sucrose content was observed at the R3 stage under the H1, H3, and H4 treatments, whereas under the H2 treatment, the highest value occurred at the R4 stage. In HN44, sucrose content under the H3 and H4 treatments also showed a trend of first increasing and then decreasing over time, whereas under the H1 treatment, it decreased gradually. Flowering-stage MC application significantly affected leaf sucrose content in HN65 at the R2, R3, R4, and R6 stages. Flowering-stage MC application also significantly affected leaf sucrose content in HN44 at the R2, R3, R4, and R6 stages. With increasing MC concentration, sucrose content in the treatment groups first decreased and then increased at the R2 and R3 stages, whereas at the R4 and R6 stages, it first increased and then decreased.

##### Effects of MC Application at Both the Seedling and Flowering Stages on Sucrose Content in Soybean

As shown in Table 11, sucrose content in HN65 under the F1, F2, F3, and F4 treatments showed a pattern of first increasing and then decreasing from R2 to R6, with the highest values observed at the R3 stage. In HN44, sucrose content under the FCK, F1, F2, and F3 treatments also showed a trend of first increasing and then decreasing over time. The highest values occurred at the R3 stage under the FCK and F3 treatments, whereas under the F1 and F2 treatments, the highest values were observed at the R4 stage. Combined application at the seedling and flowering stages significantly affected leaf sucrose content in HN65 at the R2, R3, R4, and R6 stages. With increasing MC concentration, sucrose content in the treatment groups first increased and then decreased at the R2, R3, and R4 stages, with the highest values observed under the F2, F3, and F2 treatments, respectively. At the R2 stage, sucrose content in all treatment groups was lower than that in FCK. Combined application at the seedling and flowering stages also significantly affected leaf sucrose content in HN44 at the R2, R3, R4, and R6 stages. With increasing MC concentration, sucrose content in the treatment groups first increased and then decreased at the R2, R3, R4, and R6 stages, with the highest values observed under the F3, F3, F2, and F2 treatments, respectively. At the R3 stage, sucrose content in all treatment groups was lower than that in FCK.

#### 2.3.4. Interaction Analysis of Application Timing and Sampling Stage on Main Carbohydrate Contents in Soybean Under Different MC Concentrations

As shown in Table 12, the significance (Sig.) values of the F-tests for the effects of application timing, MC concentration, and their interaction on starch, soluble sugar, and sucrose contents in both HN65 and HN44 were all greater than 0.05. Overall, the two-way ANOVA revealed that the interaction between application timing and MC concentration on starch, soluble sugar, and sucrose contents was not significant in either cultivar (Table 12). This indicates that the effects of MC concentration and application timing on leaf carbohydrate contents were largely independent of each other.

### 2.4. Effects of Different MC Concentrations on Soybean Yield

#### 2.4.1. Effects of Different MC Concentrations on Soybean Yield Components

As shown in Table 13, application of different concentrations of MC significantly affected seed weight per plant, pod number per plant, and seed number per plant in HN65. Under seedling-stage application, seed weight per plant ranked as M3 > M4 > MCK > M2 > M1. Under flowering-stage application, seed weight per plant ranked as HCK > H4 > H2 > H3 > H1. Under combined application at the seedling and flowering stages, seed weight per plant ranked as F4 > F1 > F3 > FCK > F2. As shown in Table 13, application of different MC concentrations significantly affected seed weight per plant, pod number per plant, seed number per plant, seeds per pod, and shriveled seed rate in HN44. Under seedling-stage application, seed weight per plant ranked as M2 > M4 > M3 > MCK > M1. Under the flowering-stage application, seed weight per plant ranked as H1 > H4 > H3 > H2 > HCK. Under combined application at the seedling and flowering stages, seed weight per plant ranked as F4 > F3 > FCK > F2 > F1. For HN44 at the flowering stage, although seed number per plant decreased under H3 (200 mg/L) and H4 (400 mg/L) treatments, grain yield per plant increased compared to HCK. This suggests that yield improvement was primarily driven by increased individual seed weight rather than seed number, as also reflected by the reduced empty grain rate in these treatments.

#### 2.4.2. Interaction Analysis of Application Timing and MC Concentration on Soybean Yield Under Different MC Concentrations

As shown in Table 14, the significance (Sig.) values of the F-tests for the effect of MC concentration on seeds per pod in HN65 and for the interaction between application timing and MC concentration on seed number per plant in HN44 were greater than 0.05. These results indicate that MC concentration had no significant effect on seeds per pod in HN65 and that the interaction between application timing and concentration had no significant effect on seed number per plant in HN44. In contrast, significant effects were observed for the remaining yield-related traits.

### 2.5. Correlation Analysis

**Figure 14:**
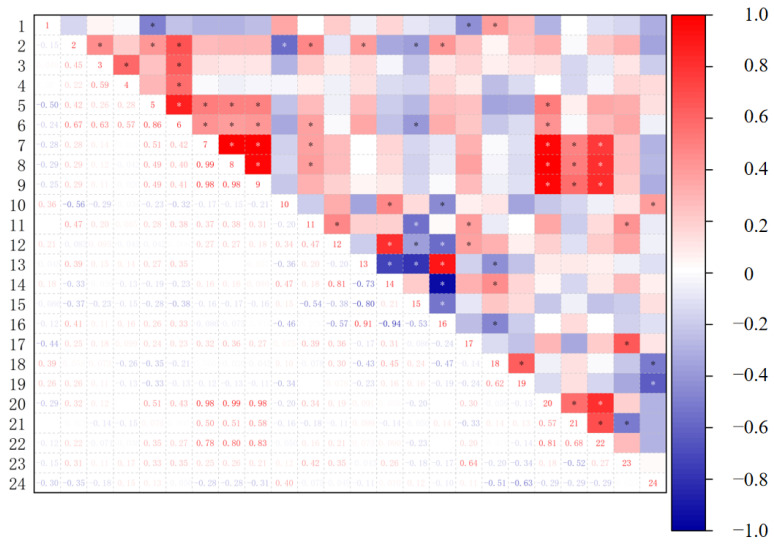
Correlation analysis of MC with indicators of soybean growth and photosynthetic characteristics. Note: The correlation significance level is: * *p* < 0.05, 1–24 in the figure represent dry matter accumulation of root, dry matter accumulation of stem, dry matter accumulation of leaf, dry matter accumulation of petiole, dry matter accumulation of pod, dry matter accumulation of whole plant, Pn, Gs, Tr, Ci, Fv/Fm, qL, NPQT, Phi2, PhiNO, PhiNPQ, starch, soluble sugar, sucrose, yield per plant, pods per plant, seeds per plant, seeds per pod, and empty grain rate.

As illustrated in Figure 14, the dry matter accumulation of the entire plant exhibited a significant positive correlation with the dry matter accumulation of the stem, leaf, petiole, and pod, as well as with the photosynthetic parameters Pn, Gs, Tr, Fv/Fm, yield per plant, pods per plant, and seeds per plant (*p* < 0.05). Starch demonstrated significant positive correlations with Fv/Fm, qL, and seeds per pod. Additionally, soluble sugar showed significant positive correlations with Phi2 and sucrose. Yield per plant was significantly positively correlated with the dry matter accumulation of the pod, the overall dry matter accumulation of the plant, Pn, Gs, Tr, pods per plant, and seeds per plant. Furthermore, pods per plant exhibited significant positive correlations with Pn, Gs, Tr, yield per plant, and seeds per plant. Lastly, seeds per plant revealed significant positive correlations with Pn, Gs, Tr, yield per plant, and pods per plant.

## 3. Discussion

### 3.1. Effects of MC on Dry Matter Accumulation in Soybean

The level of dry matter reflects the growth and yield of plants. Luo reported that MC alters dry matter accumulation and partitioning in plants. The results of the present study further support this conclusion [39]. Our results corroborate previous findings that MC alters dry matter accumulation and partitioning and further reveal that this effect is strongly dependent on application timing, concentration, and cultivar. Seedling-stage MC application generally promoted a transient shift of assimilates toward roots in HN65 at early stages, but this allocation pattern reversed at later stages, favoring leaf and petiole development. This shift suggests a dynamic rebalancing of source–sink relationships in response to MC-induced reduction in biomass accumulation. Notably, the inhibitory effect of MC on dry matter accumulation was temporary; plants exhibited a compensatory ‘rebound’ in growth during subsequent stages, particularly under moderate concentrations (200 mg/L). The dynamic pattern of dry matter accumulation observed in HN65 under seedling-stage MC application is consistent with the findings of Qu, who reported a similar trend in total dry matter accumulation of HN65 in response to MC spraying. This biphasic response may reflect a temporary promotion of vegetative growth shortly after MC application, followed by a reallocation of assimilates toward reproductive organs as the plant enters later developmental stages. The transient nature of this effect suggests that the impact of MC on dry matter partitioning is not sustained throughout the entire growing season, and the timing of peak accumulation may be critical for determining yield outcomes. The concordance between our results and those of Qu reinforces the notion that HN65 exhibits a characteristic response pattern to MC, with moderate concentrations (200 mg/L) producing the most favorable balance between vegetative growth and reproductive development [40]. This compensatory response is likely attributed to the feedback regulation of endogenous gibberellin (GA) homeostasis. Mepiquat chloride is known to inhibit GA biosynthesis. The subsequent growth recovery could involve a compensatory upregulation of GA synthesis or signaling pathways, a mechanism that has been reported in other plant species following the removal of growth retardants [41], and the subsequent recovery could involve upregulation of GA synthesis or signaling pathways [42]. The magnitude of this rebound varied between HN44 and HN65, suggesting cultivar-specific sensitivity in GA metabolism or downstream growth responses.

### 3.2. Effects of MC on Photosynthetic Characteristics in Soybean

Tian’s study demonstrated that under suitable conditions, MC can synergistically enhance photosynthetic capacity, stomatal conductance, and transpiration rate, whereas Ci was not significantly affected [43]. These findings are generally consistent with the results of the present study. Our results demonstrate that MC enhances photosynthetic performance through both stomatal and non-stomatal mechanisms, with the relative contribution of each depending on concentration and cultivar. The increase in Pn under moderate MC concentrations (100–200 mg/L) was accompanied by parallel increases in Gs and Tr, while Ci remained relatively stable. This suggests that the enhanced photosynthesis was primarily driven by improved stomatal opening and CO_2_ diffusion, rather than by changes in intercellular CO_2_ availability [19]. However, under high MC concentrations (400 mg/L), the decrease in Pn without a corresponding drop in Ci implies the occurrence of non-stomatal limitations, possibly due to damage to PSII reaction centers or reduced RuBisCO activity. Chlorophyll fluorescence parameters provided further insight into the non-stomatal effects. The increase in Fv/Fm and ΦII under optimal MC treatments indicates improved photochemical efficiency and reduced photoinhibition. Concurrently, the elevated NPQ and ΦNPQ in HN65 at the R4 stage suggest an enhanced capacity for photoprotective energy dissipation, which may help alleviate oxidative damage to the photosynthetic apparatus under high light conditions [44]. The contrasting responses of HN44 and HN65 to flowering-stage MC application—with HN65 showing decreased Fv/Fm and HN44 showing increased Fv/Fm—point to genotypic differences in photoprotective mechanisms, such as the regulation of the xanthophyll cycle or antioxidant enzyme activity.

### 3.3. Effects of MC on Photosynthates in Soybean

A previous study has shown that MC can improve the carbohydrate source–sink relationship in cotton, slightly reducing starch reserves in stems while increasing crop yield [45]. Another study reported that, in late-sown crops, MC application was negatively correlated with photosynthetic rate; specifically, as MC concentration increased, photosynthesis declined, whereas starch and sucrose contents in leaves gradually increased, suggesting that MC may inhibit the export of assimilates from source leaves to sink organs, leading to feedback inhibition of photosynthesis [46]. Ahmad reported that seedling-stage MC application in cotton promoted the accumulation of starch, soluble sugars, and sucrose in leaves at different growth stages, which is in agreement with the present findings [47]. In addition, seedling-stage application and combined application at the seedling and flowering stages increased leaf soluble sugar content in HN44 at the R6 stage, suggesting that MC can maintain higher carbohydrate availability during late reproductive stages, which may benefit seed filling. Dong further showed through proteomic analysis that MC can upregulate proteins involved in gluconeogenesis and starch metabolism, thereby increasing soluble sugar content in soybean leaves [48].

A key question arising from our results is whether MC promotes the accumulation of photoassimilates in source leaves or enhances their export to sink organs. Our findings suggest that the answer is stage-dependent. The application of MC at the seedling stage increased leaf starch and sucrose contents in both cultivars during the early stages (R1–R2), indicating a transient accumulation of carbohydrates in source leaves. This accumulation may result from either enhanced photosynthetic carbon fixation or, more likely, a temporary inhibition of phloem loading and export, as proposed by Luo and Tang [23,39]. However, this accumulation did not lead to feedback inhibition of photosynthesis, as Pn remained elevated or recovered quickly in most treatments. This contrasts with the findings of Tung et al. in late-planted cotton, where MC-induced sugar accumulation was associated with sustained photosynthetic suppression [24]. The discrepancy may be attributed to differences in crop species, planting density, or the timing of MC application relative to the sink–source transition. Notably, the stimulatory effect of MC on leaf carbohydrate content was more pronounced when applied at the seedling stage than at the flowering stage. This stage-dependent response likely reflects the higher metabolic activity and greater sink demand of leaves during vegetative growth, which may facilitate the subsequent mobilization of stored carbohydrates to developing pods during reproductive stages [49]. In HN44, the increased soluble sugar content at R6 under seedling-stage and combined applications suggests that MC can sustain carbohydrate availability during late reproductive stages, thereby benefiting seed filling.

Beyond the physiological responses observed in photosynthesis and carbohydrate metabolism, the regulatory effects of MC on soybean growth and yield are likely mediated by upstream hormonal and ionic signaling networks. Plants are complex systems in which development is governed by dynamic interactions not only among organs but also among distinct cell types within a single organ [50]. The local balance of phytohormones—particularly gibberellins, cytokinins, and abscisic acid—plays a critical role in integrating environmental and developmental cues [51]. MC, as a gibberellin biosynthesis inhibitor, may perturb this hormonal equilibrium, triggering compensatory adjustments in other hormone pathways that collectively modulate cell expansion, division, and differentiation [52]. Furthermore, ion homeostasis at the cellular level exerts a direct influence on cell wall extensibility and turgor-driven expansion, processes that are fundamental to organ growth and architecture [53]. The cultivar-specific responses observed between HN44 and HN65 may therefore reflect inherent differences in their hormonal setpoints, ion transport efficiencies, or epigenetic regulation of stress-responsive genes. Future investigations at the transcriptomic, metabolomic, epigenomic, and interactomic levels—specifically, the systematic study of protein–protein and protein–metabolite interactions that connect molecular networks to physiological phenotypes—are warranted to elucidate the molecular circuitry through which MC orchestrates these multi-scale regulatory networks [54]. Integrating interactomics with spatial in situ approaches will enable researchers to map these regulatory networks within specific cell types and developmental contexts, thereby providing a more comprehensive understanding of MC’s mode of action in soybean.

### 3.4. Effects of MC on Soybean Yield

Meng reported that MC altered endogenous hormone levels, delayed cotton leaf abscission, increased boll opening rate, and ultimately improved yield [55]. The effect of MC on yield is closely related to cultivar characteristics, application concentration, and application timing. Soybean yield is determined mainly by pod number per plant, seed number per plant, seed weight, and the shriveled seed rate. Previous studies have shown that MC can increase soybean yield by increasing pod number and seed number per plant [32]. However, the effects of MC are not always consistent and may vary with genotype and treatment regime. Tung reported in cotton that leaf area and leaf area duration decreased sharply with increasing MC concentration, resulting in a gradual decline in lint yield [56]. Conversely, Chen found that MC increased boll number per unit area, retained more bolls in the middle and upper canopy, and thereby increased yield [57]. Similarly, Novakoski reported that the yield response to MC differed among soybean cultivars, which is consistent with the cultivar-specific responses observed in the present study [58].

One of the most striking findings of this study is the cultivar-specific yield response to the timing of MC application. In HN44, the application of MC (50–200 mg/L) at the flowering stage significantly increased the grain yield per plant, primarily through enhanced individual seed weight and a reduced rate of shriveled seeds, despite a slight reduction in the number of seeds per plant under some treatments. This suggests that applying MC at flowering improves the efficiency of assimilate partitioning to the remaining seeds, likely by reducing competition among developing pods and seeds [32,59]. In contrast, HN65 exhibited a more favorable response to seedling-stage MC application (200–400 mg/L), which increased both the number of seeds per plant and seed weight. The positive response of HN65 to early MC application may be attributed to its greater capacity for compensatory growth following the initial suppression of vegetative development, thereby allowing for enhanced pod set and seed filling during later stages. The contrasting responses of HN44 and HN65 can be explained by genotypic differences in endogenous hormone balance, particularly in the dynamics of gibberellins (GAs) and cytokinins, which are known to regulate floral development and seed set [9,26]. HN44, potentially possessing higher GA levels during flowering, may benefit from MC-induced GA suppression, reallocating resources from vegetative to reproductive growth. Conversely, HN65 may have a narrower optimal concentration window and greater sensitivity to MC during the reproductive phase, making seedling-stage application a safer and more effective option. From an agronomic perspective, these findings underscore the importance of tailoring MC application strategies to specific cultivars. For HN65, a single seedling-stage application or a combined seedling and flowering application of 200–400 mg/L is recommended. For HN44, a flowering-stage application of 50–200 mg/L appears to be more effective. This cultivar-specific recommendation represents a significant advancement over the current one-size-fits-all approach to MC application in soybean production.

In addition to the physiological and yield-related parameters examined in this study, future research should also investigate the effects of MC on leaf and stem morphology, including leaf area, specific leaf weight, stem diameter, and internode length. These morphological traits are closely linked to canopy architecture, light interception, and mechanical support (lodging resistance), and may contribute to the cultivar-specific yield responses observed here. A comprehensive analysis integrating morphological, physiological, and molecular data would provide a more holistic understanding of MC’s mode of action in soybean.

## 4. Materials and Methods

### 4.1. Experimental Materials

To further investigate the effects of MC on soybean growth and development, HN44 and HN65 were selected as the experimental materials in the present study. Both cultivars were bred by the Soybean Research Institute, Heilongjiang Academy of Agricultural Sciences. The experimental soil was collected from the 0–20 cm topsoil layer of a field previously cultivated with maize. The soil type is black soil, and its texture is clay loam. The bulk density of the soil from 0 to 20 cm is 1.25 g/cm^3^. The basic physicochemical properties of the soil are shown in Table 15. This experiment was conducted at the experimental station of Northeast Agricultural University (126°43′ E, 45°44′ N) located in Xiangfang District, Harbin City, Heilongjiang Province. The climate is a temperate continental climate, with an average annual precipitation of 423 mm. The MC used in this study was purchased from Hebei Guoxin Nongnong Biotechnology Co., Ltd (Cangzhou, China). The product was supplied as a soluble powder containing 98% active ingredient.

### 4.2. Experimental Design

The experiment was conducted as a pot trial. Plastic pots with a top diameter of 35 cm, a bottom diameter of 22 cm, and a height of 30 cm were used. Six drainage holes, each 1 cm in diameter, were made in the bottom of each pot, and the pots were lined with nylon mesh to ensure proper drainage. Each pot was filled with 16 kg of soil. The pots were placed in 30 cm deep trenches, and the spaces between pots were backfilled with soil. Pots were arranged in pairs, with 40 cm spacing between rows.

At sowing, eight soybean seeds with diameters ranging from 6.5 to 7.0 mm were sown in each pot. The seeds were treated with fludioxonil + metalaxyl-M prior to sowing. Basal fertilizer was applied at rates of 0.5 g potassium sulfate (K_2_O, 50%) and 1.0 g diammonium phosphate (N, 18%; P_2_O_5_, 46%) per pot. When the first trifoliolate leaf had fully expanded, the seedlings were thinned to three plants per pot. The pot experiment was conducted outdoors under natural light conditions. During the soybean growing season (May to September), the average daily temperature was approximately 20.5 °C, with a diurnal range of 15.6 °C to 25.7 °C. The average daylength was approximately 15 h. All pots were watered daily to maintain soil moisture at around 70–80% of field capacity. The precipitation during the growing season was 539.5 mm, with the major precipitation (88.28%) occurring from June to August.

The experiment employed a completely randomized design with three biological replicates per treatment. Each replicate consisted of three pots, and each pot contained three plants, resulting in nine plants per treatment per replicate. Pots were rotated weekly to minimize positional effects. All measurements were taken on the three plants within each pot, and the pot mean was used as the experimental unit for statistical analysis. The experiment included three application stages and four MC concentrations (50, 100, 200, and 400 mg/L), with water spray used as the control (CK). The application regimes were as follows: (1) application at the seedling stage (V3), followed by a second spray 3 d later; (2) application at the beginning flowering stage (R1), followed by a second spray 3 d later; and (3) application at both the seedling stage and the beginning flowering stage (V3 + R1), with two sprays applied at each stage, for a total of four applications. MC solution was sprayed uniformly onto all leaves. An electric sprayer was used to uniformly apply the MC solution onto all leaves, ensuring that the solution was sprayed until it began to drip, which typically requires approximately 50 milliliters per pot for each application, which corresponded to approximately 16.7 mL per plant (3 plants per pot). Based on the pot surface area (0.096 m^2^), this was equivalent to approximately 520 L ha^−1^ of spray solution. For treatments receiving four sprays (F series), the total solution volume applied per pot was approximately 200 mL per pot over the entire season. In total, 15 treatments were established. Detailed treatment information is presented in Table 16.

### 4.3. Measurement Indicators and Methods

#### 4.3.1. Dry Matter Weight

Sampling was conducted at the seedling stage (V3), beginning flowering stage (R1), full flowering stage (R2), beginning pod stage (R3), full pod stage (R4), seed filling stage (R6), and full maturity stage (R8). Three plants were sampled from separate pots within the same replicate to avoid pseudoreplication. At each sampling time, soybean plants were collected and separated into five components: leaves, stems, petioles, flowers/pods, and roots. For root collection, the entire soil volume of each pot was carefully washed out using a gentle stream of tap water. The pots were overturned onto a 2 mm mesh sieve, and the soil was washed away with low-pressure water to minimize root breakage. Roots were then carefully separated from the aboveground parts by cutting at the hypocotyl-root junction (the transition zone between the stem and root). After collection, roots were rinsed three times with distilled water to remove residual soil particles, blotted dry with filter paper, and placed in pre-weighed aluminum boxes for oven drying. Each treatment was tested three times in duplicate. Each component was placed in an aluminum box, heated at 105 °C for 30 min to deactivate enzymes, and then oven-dried at 65 °C to a constant weight. The dry weight of each component was determined using an analytical balance.

#### 4.3.2. Photosynthetic Characteristics

##### Determination of Main Carbohydrate Contents

Leaf samples were collected at the seedling stage (V3), initial flowering stage (R1), full flowering stage (R2), initial pod stage (R3), full pod stage (R4), seed filling stage (R6), and full maturity stage (R8). Immediately after sampling, the leaves were frozen in liquid nitrogen and then stored at −80 °C until analysis.

The contents of the major carbohydrates in leaves were determined as follows:

Sucrose content was determined by the resorcinol colorimetric method according to the procedure described by Handel [60].

Soluble sugar content was determined by the anthrone colorimetric method according to the procedure described by Yemm [61].

Starch content was determined by the anthrone colorimetric method according to the procedure described by McCready [62].

##### Determination of Photosynthesis-Related Parameters

Chlorophyll fluorescence parameters were measured using a MultispeQ V2.0 instrument from Beijing Huinuoruide Technology Co., Ltd (Beijing, China). The measured fluorescence parameters included the maximum quantum efficiency of PSII (Fv/Fm), qL, NPQ, effective quantum yield of PSII (ΦII), quantum yield of regulated non-photochemical energy dissipation in PSII (ΦNPQ), and quantum yield of non-regulated energy dissipation in PSII (ΦNO). Gas-exchange parameters were measured using a portable photosynthesis system (Yaxin-1101, Yaxinliyi Science and Technology Co., Ltd., Beijing, China). The measured photosynthetic parameters included Pn, intercellular CO_2_ concentration (Ci), Gs, and Tr. Measurements were taken from the lamina area between the leaf margin and the main vein. All measurements were performed at the R1, R2, and R4 growth stages. R8 growth stage represents full maturity, where leaves have senesced, making photosynthetic measurements unreliable.

All gas-exchange and chlorophyll fluorescence measurements were performed between 9:00 AM and 11:30 AM on clear days. For gas-exchange parameters, the leaf chamber was set to maintain a photosynthetically active radiation (PAR) of 1200 ± 50 μmol m^−2^ s^−1^, a reference CO_2_ concentration of 400 ± 10 μmol mol^−1^, and a leaf temperature of 28 ± 2 °C. The relative humidity inside the chamber was maintained at 60–70%.

Photosynthetic measurements were focused on the R1, R2, and R4 stages because these periods represent the critical transition from vegetative to reproductive growth and the peak pod development phase, during which photosynthetic capacity is most determinant for yield formation. By the R6 stage (seed filling), leaf senescence progresses rapidly, and the reliability of gas-exchange measurements under consistent conditions diminishes, making it less suitable for comparative analysis.

#### 4.3.3. Yield Measurement

At soybean maturity, six pots were selected for yield component analysis. Seed weight per plant, pod number per plant, and seed number per plant were measured. The number of seeds per pod and the shriveled seed rate were then calculated.

### 4.4. Statistical Analysis

Analysis of variance (ANOVA) was performed for stem growth, dry matter accumulation, photosynthesis-related parameters, and yield using IBM SPSS Statistics for Windows, Version 21.0 (IBM Corp., Armonk, NY, USA). Mean comparisons were conducted using the Waller–Duncan test at the 0.05 significance level. Figures were generated using Origin 2021.

## 5. Conclusions

This study demonstrates that the yield response of soybean to MC is cultivar-specific and highly dependent on application timing. The underlying mechanism involves stage-dependent modulation of photosynthetic efficiency (particularly PSII photochemistry) and carbohydrate partitioning. Our findings provide a physiological basis for developing cultivar-tailored MC application strategies, recommending a single seedling-stage application (200–400 mg/L) for HN65 and a flowering-stage application (50–200 mg/L) for HN44 to maximize yield.

## Figures and Tables

**Table 1:** Analysis of the interaction between spraying and sampling of different concentrations of MC on dry matter accumulation of soybean.

Variety	Site	Spraying Period		Treatment Concentration		Spraying Period × Treatment Concentration	
		F	Sig	F	Sig	F	Sig
HN65	Leaf	0.4	0.67	22.25	<0.01	1.21	0.3
	Stem	1.51	0.23	17.79	<0.01	1.1	0.37
	Petiole	1.1	0.34	22.85	<0.01	2.47	0.02
	Root	0.89	0.41	1.21	0.31	3.41	<0.01
	Flower/Pod	0.21	0.81	5.18	<0.01	6.15	<0.01
HN44	Leaf	21.64	<0.01	13.76	<0.01	2.95	<0.01
	Stem	37.57	<0.01	0.72	0.58	4.29	<0.01
	Petiole	10.52	<0.01	17.47	<0.01	3.76	<0.01
	Root	17.13	<0.01	19.84	<0.01	12.25	<0.01
	Flower/Pod	71.48	<0.01	16.56	<0.01	10.67	<0.01

**Table 2:** Analysis of the interaction between spraying and sampling of different concentrations of MC on soybean photosynthetic parameters.

Variety	Index	Spraying Period		Treatment Concentration		Spraying Period × Treatment Concentration	
		F	Sig	F	Sig	F	Sig
HN65	Pn	62.89	<0.01	10.76	<0.01	7.65	<0.01
	Gs	55.72	<0.01	8.44	<0.01	5.71	<0.01
	Tr	48.17	<0.01	13.87	<0.01	5.55	<0.01
	Ci	1.19	0.31	1.85	0.13	0.38	0.93
	Fv/Fm	9.1	<0.01	3.59	<0.01	4.57	<0.01
	qL	7.33	<0.01	6.78	<0.01	2.42	0.02
	NPQ	7.16	<0.01	11.3	<0.01	3.63	<0.01
	Phi2	0.55	0.58	7.36	<0.01	2.05	0.05
	PhiNO	22.76	<0.01	14.29	<0.01	5.24	<0.01
	PhiNPQ	4.27	0.02	10.04	<0.01	2.25	0.03
HN44	Pn	53.3	<0.01	56.23	<0.01	17.22	<0.01
	Gs	44.52	<0.01	32.8	<0.01	15.02	<0.01
	Tr	46.48	<0.01	31.49	<0.01	13.33	<0.01
	Ci	1.89	0.16	0.35	0.84	0.32	0.96
	Fv/Fm	10.03	<0.01	1.49	0.21	13.51	<0.01
	qL	10.22	<0.01	5.25	<0.01	9.33	<0.01
	NPQ	31.04	<0.01	59.44	<0.01	9.98	<0.01
	Phi2	1.47	0.24	32	<0.01	2.79	<0.01
	PhiNO	67.68	<0.01	12.19	<0.01	9.77	<0.01
	PhiNPQ	9.9	<0.01	52.41	<0.01	2.78	<0.01

**Table 3:** Effects of spraying different concentrations of MC on soybean starch content at the seedling stage (mg/g·DW).

Variety	Treatments	R1	R2	R3	R4	R6
HN65	MCK	41.51 ± 2.98 c	73.21 ± 2.15 a	40.27 ± 2.38 c	63.30 ± 2.47 b	59.80 ± 5.52 b
	M1	54.66 ± 4.40 b	72.55 ± 4.24 a	59.39 ± 4.10 a	73.72 ± 4.61 a	72.80 ± 5.63 a
	M2	67.51 ± 3.10 a	73.23 ± 3.74 a	33.67 ± 2.79 d	64.02 ± 3.58 b	51.46 ± 0.60 b
	M3	59.34 ± 3.48 b	72.11 ± 6.44 a	37.25 ± 3.07 cd	74.85 ± 5.38 a	56.46 ± 3.50 b
	M4	57.09 ± 2.51 b	63.13 ± 5.02 a	51.36 ± 2.41 b	76.56 ± 2.81 a	77.19 ± 4.69 a
HN44	MCK	62.65 ± 2.45 a	66.18 ± 4.24 ab	43.40 ± 4.47 b	45.13 ± 2.98 b	46.50 ± 4.11 ab
	M1	54.76 ± 2.93 ab	61.22 ± 4.79 b	46.08 ± 1.73 b	50.33 ± 2.38 b	43.10 ± 1.69 ab
	M2	60.29 ± 3.95 ab	74.93 ± 2.94 a	48.30 ± 5.84 b	51.40 ± 8.68 ab	40.29 ± 3.44 ab
	M3	54.47 ± 4.78 b	72.77 ± 5.17 ab	67.43 ± 4.60 a	62.27 ± 0.99 a	39.01 ± 1.57 b
	M4	43.46 ± 3.68 c	71.75 ± 5.83 ab	65.01 ± 2.00 a	51.73 ± 4.00 ab	52.11 ± 8.78 a

Note: Different letters in the table indicate significant difference at the 0.05 level among treatments on the same date (*p* < 0.05).

**Table 4:** Effects of spraying different concentrations of MC on starch content of soybean at flowering stage (mg/g·DW).

Variety	Treatments	R2	R3	R4	R6
HN65	HCK	72.21 ± 1.59 a	40.60 ± 0.24 b	61.97 ± 1.64 a	59.47 ± 1.11 a
	H1	60.23 ± 4.02 b	27.00 ± 4.13 c	56.35 ± 5.58 a	51.26 ± 4.58 b
	H2	72.87 ± 3.79 a	32.75 ± 2.82 c	43.58 ± 3.48 b	43.72 ± 1.34 cd
	H3	73.23 ± 2.98 a	66.84 ± 3.11 a	60.36 ± 4.71 a	43.41 ± 2.15 d
	H4	80.30 ± 6.52 a	40.98 ± 3.15 b	64.67 ± 3.33 a	49.41 ± 2.60 bc
HN44	HCK	65.51 ± 1.39 b	42.73 ± 1.23 c	44.13 ± 0.61 b	45.17 ± 1.58 a
	H1	95.53 ± 8.81 a	75.70 ± 2.03 a	68.06 ± 2.56 a	32.01 ± 3.47 bc
	H2	43.51 ± 1.78 cd	26.71 ± 1.82 d	37.20 ± 4.67 c	37.72 ± 3.83 b
	H3	42.27 ± 3.40 d	23.18 ± 1.65 e	37.74 ± 3.10 c	33.76 ± 2.75 bc
	H4	52.38 ± 2.11 c	47.79 ± 0.06 b	38.48 ± 2.23 bc	27.85 ± 2.72 c

Note: Different letters in the table indicate significant difference at the 0.05 level among treatments on the same date (*p* < 0.05).

**Table 5:** Effects of different concentrations of MC on soybean starch content by seedling + flower spraying (mg/g·DW).

Variety	Treatments	R1	R2	R3	R4	R6
HN65	FCK	41.51 ± 2.98 c	70.55 ± 2.77 ab	40.27 ± 1.72 c	60.63 ± 2.33 b	59.80 ± 2.19 a
	F1	54.66 ± 4.40 b	78.92 ± 2.97 a	52.44 ± 5.44 b	51.72 ± 4.51 c	38.47 ± 1.17 c
	F2	67.51 ± 3.10 a	76.37 ± 2.82 a	66.60 ± 4.77 a	51.89 ± 1.78 c	51.80 ± 2.38 b
	F3	59.34 ± 3.48 b	60.81 ± 5.29 bc	53.97 ± 2.33 b	58.61 ± 0.82 b	61.64 ± 3.87 a
	F4	57.09 ± 2.51 b	51.57 ± 11.32 c	53.59 ± 2.25 b	71.03 ± 2.08 a	65.16 ± 5.76 a
HN44	FCK	62.65 ± 2.45 a	64.51 ± 2.59 d	43.73 ± 1.10 c	44.80 ± 1.20 c	45.50 ± 2.02 b
	F1	54.76 ± 2.93 ab	83.23 ± 6.15 c	45.46 ± 1.67 bc	37.34 ± 1.02 d	28.36 ± 1.36 d
	F2	60.29 ± 3.95 ab	96.32 ± 8.48 b	41.83 ± 2.69 c	65.38 ± 3.44 a	58.56 ± 4.25 a
	F3	54.47 ± 4.78 b	101.16 ± 3.00 ab	51.06 ± 5.66 b	56.66 ± 1.68 b	64.23 ± 3.66 a
	F4	43.46 ± 3.68 c	108.44 ± 3.76 a	69.67 ± 2.53 a	46.43 ± 3.04 c	36.34 ± 1.84 c

Note: Different letters in the table indicate significant difference at the 0.05 level among treatments on the same date (*p* < 0.05).

**Table 6:** Effects of spraying different concentrations of MC on soluble sugar content of soybean at the seedling stage (mg/g·DW).

Variety	Treatments	R1	R2	R3	R4	R6
HN65	MCK	194.50 ± 6.69 a	230.13 ± 8.32 a	324.41 ± 11.63 b	299.93 ± 12.30 b	288.96 ± 9.30 a
	M1	207.51 ± 7.68 a	210.79 ± 3.32 b	369.52 ± 5.02 a	320.26 ± 2.78 a	286.65 ± 6.13 a
	M2	195.66 ± 4.21 a	204.99 ± 7.29 b	245.28 ± 4.49 d	297.66 ± 5.83 b	273.39 ± 5.35 ab
	M3	195.60 ± 5.04 a	198.96 ± 8.50 b	274.76 ± 5.38 c	266.35 ± 2.23 d	262.10 ± 8.43 b
	M4	179.72 ± 6.42 b	198.40 ± 5.44 b	274.96 ± 7.67 c	281.20 ± 2.96 c	268.20 ± 5.66 b
HN44	MCK	243.31 ± 3.44 c	287.56 ± 3.92 b	391.07 ± 6.09 a	279.12 ± 4.75 b	274.12 ± 3.75 b
	M1	217.78 ± 6.35 d	219.73 ± 8.75 d	372.61 ± 9.14 a	278.81 ± 10.50 b	277.14 ± 11.28 b
	M2	238.67 ± 8.59 c	274.76 ± 4.14 b	381.17 ± 9.58 a	292.40 ± 8.87 b	291.16 ± 13.73 ab
	M3	266.03 ± 5.14 b	306.32 ± 2.54 a	382.82 ± 13.51 a	351.58 ± 11.07 a	315.50 ± 12.38 a
	M4	297.72 ± 6.37 a	256.57 ± 10.74 c	317.80 ± 3.65 b	348.60 ± 12.63 a	291.70 ± 16.88 ab

Note: Different letters in the table indicate significant difference at the 0.05 level among treatments on the same date (*p* < 0.05).

**Table 7:** Effects of spraying different concentrations of MC on soluble sugar content of soybean at flowering stage (mg/g·DW).

Variety	Treatments	R2	R3	R4	R6
HN65	HCK	231.60 ± 4.55 a	320.73 ± 6.13 a	294.54 ± 3.06 a	290.24 ± 6.71 b
	H1	173.06 ± 10.23 b	334.02 ± 8.46 a	252.11 ± 10.39 b	277.00 ± 9.84 bc
	H2	259.35 ± 4.13 c	301.57 ± 9.03 b	252.48 ± 0.82 b	285.91 ± 6.18 bc
	H3	119.48 ± 1.47 d	258.58 ± 8.40 c	243.64 ± 12.71 b	316.92 ± 9.91 a
	H4	131.28 ± 3.75 d	263.18 ± 9.90 c	256.09 ± 3.21 b	260.77 ± 8.38 c
HN44	HCK	289.27 ± 2.62 c	394.44 ± 4.53 a	280.12 ± 2.73 b	272.65 ± 5.91 a
	H1	250.35 ± 7.76 d	257.24 ± 5.96 e	291.86 ± 6.48 b	225.20 ± 6.98 c
	H2	259.35 ± 4.13 d	301.57 ± 9.03 c	321.45 ± 6.37 a	285.91 ± 6.18 a
	H3	311.65 ± 4.77 b	325.15 ± 4.63 b	317.00 ± 4.53 a	259.10 ± 5.30 b
	H4	380.89 ± 5.37 a	282.31 ± 3.36 d	292.69 ± 8.34 b	246.50 ± 7.25 b

Note: Different letters in the table indicate significant difference at the 0.05 level among treatments on the same date (*p* < 0.05).

**Table 8:** Effects of different concentrations of MC on soluble sugar content of soybean by seedling + flower spraying (mg/g·DW).

Variety	Treatments	R1	R2	R3	R4	R6
HN65	FCK	194.50 ± 6.69 a	229.85 ± 2.55 a	321.07 ± 4.72 a	298.26 ± 18.20 a	286.93 ± 7.22 b
	F1	207.51 ± 7.68 a	158.93 ± 7.05 c	301.00 ± 6.02 b	235.09 ± 1.88 b	230.58 ± 8.39 d
	F2	195.66 ± 4.21 a	160.10 ± 6.78 c	240.84 ± 10.31 c	248.30 ± 5.71 b	257.98 ± 5.84 c
	F3	195.60 ± 5.04 a	185.67 ± 10.14 b	216.70 ± 6.85 d	204.78 ± 7.99 c	308.17 ± 6.91 a
	F4	179.72 ± 6.42 b	200.31 ± 6.99 b	228.60 ± 10.16 cd	203.02 ± 9.66 c	276.75 ± 10.22 b
HN44	FCK	243.31 ± 3.44 c	283.34 ± 4.84 c	388.24 ± 4.62 a	275.79 ± 10.36 c	272.18 ± 4.36 c
	F1	217.78 ± 6.35 d	271.31 ± 7.48 c	309.05 ± 11.10 b	301.10 ± 2.47 a	306.46 ± 9.40 b
	F2	238.67 ± 8.59 c	375.53 ± 1.07 b	308.81 ± 7.87 b	307.86 ± 4.42 a	350.43 ± 7.82 a
	F3	266.03 ± 5.14 b	394.37 ± 6.93 a	301.88 ± 6.46 bc	296.00 ± 9.02 ab	306.26 ± 6.98 b
	F4	297.72 ± 6.37 a	384.12 ± 9.91 ab	292.50 ± 2.89 c	285.35 ± 4.96 bc	301.01 ± 8.98 b

Note: Different letters in the table indicate significant difference at the 0.05 level among treatments on the same date (*p* < 0.05).

**Table 9:** Effects of spraying different concentrations of MC on the sucrose content of soybean at the seedling stage (mg/g·DW).

Variety	Treatments	R1	R2	R3	R4	R6
HN65	MCK	24.79 ± 0.57 d	29.19 ± 1.40 bc	27.90 ± 2.48 b	24.68 ± 2.04 b	20.74 ± 0.30 a
	M1	29.07 ± 1.49 ab	30.99 ± 2.05 bc	33.10 ± 0.24 ab	31.64 ± 2.03 a	15.92 ± 1.07 b
	M2	30.40 ± 0.95 a	33.67 ± 3.21 b	37.57 ± 3.83 a	30.99 ± 1.56 a	15.49 ± 0.68 b
	M3	27.86 ± 0.44 bc	42.61 ± 3.05 a	36.94 ± 1.77 a	28.33 ± 1.5 ab	17.17 ± 0.68 b
	M4	26.51 ± 0.65 cd	27.03 ± 1.65 c	31.07 ± 2.55 b	31.80 ± 1.49 a	21.94 ± 1.70 a
HN44	MCK	27.73 ± 3.90 b	37.55 ± 1.94 a	38.42 ± 0.72 b	29.11 ± 1.85 d	25.24 ± 1.10 a
	M1	30.36 ± 0.97 b	35.02 ± 1.00 a	34.38 ± 2.92 b	30.55 ± 0.36 cd	24.37 ± 0.49 ab
	M2	37.47 ± 1.05 a	37.50 ± 1.95 a	37.21 ± 2.30 b	32.61 ± 1.51 bc	22.57 ± 1.28 b
	M3	31.50 ± 2.27 b	34.39 ± 0.74 a	43.72 ± 2.33 a	39.27 ± 1.75 a	17.38 ± 1.50 c
	M4	28.16 ± 2.25 b	33.93 ± 1.51 a	34.32 ± 1.71 b	35.57 ± 1.88 b	16.98 ± 1.00 c

Note: Different letters in the table indicate significant difference at the 0.05 level among treatments on the same date (*p* < 0.05).

**Table 10:** Effects of spraying different concentrations of MC on the sucrose content of soybean at the flowering stage (mg/g·DW).

Variety	Treatments	R2	R3	R4	R6
HN65	HCK	29.66 ± 1.57 a	27.15 ± 0.62 c	24.53 ± 1.16 b	21.02 ± 1.39 a
	H1	24.76 ± 1.92 b	30.26 ± 2.97 bc	27.51 ± 1.64 b	14.92 ± 0.25 d
	H2	23.94 ± 1.49 bc	29.16 ± 2.91 bc	33.57 ± 0.88 a	16.71 ± 1.11 cd
	H3	18.33 ± 1.38 d	33.42 ± 1.59 b	26.82 ± 2.38 b	20.06 ± 0.89 ab
	H4	21.08 ± 0.67 cd	37.94 ± 0.58 a	25.66 ± 0.89 b	18.56 ± 0.96 bc
HN44	HCK	38.07 ± 1.65 a	38.53 ± 1.56 a	28.96 ± 2.00 c	25.09 ± 0.20 a
	H1	39.64 ± 1.11 a	34.63 ± 1.07 b	30.21 ± 2.54 c	19.29 ± 1.11 b
	H2	37.66 ± 0.67 a	24.30 ± 1.47 d	31.01 ± 1.30 bc	19.51 ± 1.19 b
	H3	27.16 ± 2.48 b	31.01 ± 0.64 c	36.74 ± 2.13 a	14.05 ± 0.87 c
	H4	28.50 ± 2.25 b	31.52 ± 1.73 c	34.89 ± 1.03 ab	13.99 ± 0.93 c

Note: Different letters in the table indicate significant difference at the 0.05 level among treatments on the same date (*p* < 0.05).

**Table 11:** Effects of different concentrations of MC on sucrose content of soybean by seedling + flower spraying (mg/g·DW).

Variety	Treatments	R1	R2	R3	R4	R6
HN65	FCK	24.79 ± 0.57 d	29.13 ± 1.60 a	28.18 ± 0.46 b	25.28 ± 0.46 a	20.55 ± 0.85 a
	F1	29.07 ± 1.49 ab	12.73 ± 0.80 c	33.32 ± 3.02 a	23.46 ± 0.36 b	15.35 ± 1.49 b
	F2	30.40 ± 0.95 a	19.89 ± 1.82 b	33.46 ± 1.97 a	25.86 ± 1.33 a	15.23 ± 1.27 b
	F3	27.86 ± 0.44 bc	24.58 ± 0.96 b	31.66 ± 1.14 ab	26.03 ± 0.37 a	23.04 ± 1.39 a
	F4	26.51 ± 0.65 cd	21.52 ± 4.21 b	30.44 ± 0.64 ab	24.56 ± 0.62 ab	23.40 ± 2.12 a
HN44	FCK	27.73 ± 3.90 b	37.95 ± 0.11 a	38.61 ± 0.89 a	29.01 ± 1.40 d	24.99 ± 0.21 a
	F1	30.36 ± 0.97 b	26.02 ± 1.52 c	28.75 ± 1.86 c	33.70 ± 1.64 c	17.53 ± 1.52 d
	F2	37.47 ± 1.05 a	31.77 ± 2.93 b	35.16 ± 2.79 ab	41.24 ± 0.63 a	22.15 ± 1.07 b
	F3	31.50 ± 2.27 b	38.41 ± 2.01 a	38.55 ± 1.51 a	38.44 ± 1.07 b	19.89 ± 1.12 c
	F4	28.16 ± 2.25 b	34.25 ± 1.78 ab	34.04 ± 1.89 b	38.12 ± 1.71 b	10.78 ± 0.39 e

Note: Different letters in the table indicate significant difference at the 0.05 level among treatments on the same date (*p* < 0.05).

**Table 12:** Analysis of the interaction between spraying and sampling of different concentrations of MC on soybean photosynthetic parameters.

Variety	Index	Spraying Period		Treatment Concentration		Spraying Period × Treatment Concentration	
		F	Sig	F	Sig	F	Sig
HN65	Starch	21.77	<0.01	10.21	<0.01	14.42	<0.01
	Soluble Sugar	77.8	<0.01	147.61	<0.01	16.06	<0.01
	Sucrose	36.54	<0.01	8.03	<0.01	7.33	<0.01
HN44	Starch	83.7	<0.01	6.11	<0.01	80.78	<0.01
	Soluble Sugar	65.58	<0.01	138.17	<0.01	19.61	<0.01
	Sucrose	15.26	<0.01	19.35	<0.01	13.27	<0.01

**Table 13:** Effects of spraying different concentrations of MC on the yield of soybean.

Varieties	Treatments	Yield Per Plant (g)	Pods Per Plant (No.)	Seeds Per Plant (No.)	Seeds Per Pod (No.)	Empty Grain Rate (%)
HN65	MCK	17.70 ± 0.82 b	36.50 ± 4.34 ab	91.00 ± 8.46 b	2.50 ± 0.14 b	0.21 ± 0.03 a
	M1	17.44 ± 0.49 b	35.00 ± 4.05 ab	94.00 ± 9.67 ab	2.69 ± 0.05 ab	0.18 ± 0.01 b
	M2	17.45 ± 0.60 b	33.00 ± 1.67 b	92.20 ± 6.49 a	2.79 ± 0.12 a	0.14 ± 0.01 c
	M3	20.09 ± 0.46 a	39.20 ± 2.04 a	102.40 ± 5.31 ab	2.62 ± 0.13 ab	0.12 ± 0.01 cd
	M4	19.67 ± 1.44 a	38.40 ± 2.73 a	110.40 ± 7.26 a	2.89 ± 0.31 a	0.11 ± 0.01 d
	HCK	17.62 ± 1.23 a	36.80 ± 1.33 a	92.40 ± 3.26 a	2.51 ± 0.09 a	0.21 ± 0.04 a
	H1	14.26 ± 1.44 b	28.20 ± 1.94 b	73.20 ± 6.37 b	2.59 ± 0.08 a	0.20 ± 0.02 a
	H2	15.20 ± 1.30 ab	30.60 ± 2.65 b	77.80 ± 6.46 b	2.54 ± 0.04 a	0.19 ± 0.03 a
	H3	15.08 ± 2.05 b	30.80 ± 4.35 b	78.00 ± 8.65 b	2.54 ± 0.10 a	0.21 ± 0.01 a
	H4	16.46 ± 1.25 ab	32.20 ± 2.64 b	82.60 ± 5.46 b	2.57 ± 0.13 a	0.22 ± 0.03 a
	FCK	18.02 ± 0.99 b	36.30 ± 1.25 b	90.20 ± 5.34 b	2.48 ± 0.08 a	0.22 ± 0.01 ab
	F1	19.68 ± 0.83 a	43.40 ± 1.96 a	103.80 ± 7.22 a	2.39 ± 0.17 a	0.24 ± 0.03 ab
	F2	17.58 ± 0.62 b	36.00 ± 1.41 b	92.40 ± 7.66 ab	2.56 ± 0.15 a	0.18 ± 0.02 b
	F3	18.94 ± 0.95 ab	40.00 ± 4.77 ab	97.20 ± 8.13 ab	2.44 ± 0.14 a	0.21 ± 0.07 ab
	F4	19.88 ± 1.16 a	42.40 ± 3.77 a	98.80 ± 6.73 ab	2.33 ± 0.07 a	0.25 ± 0.03 a
HN44	MCK	16.14 ± 0.96 b	43.40 ± 3.67 a	99.80 ± 3.82 a	2.31 ± 0.13 a	0.13 ± 0.02 bc
	M1	13.58 ± 0.49 c	36.40 ± 3.14 b	67.80 ± 7.24 c	1.87 ± 0.24 b	0.29 ± 0.08 a
	M2	18.50 ± 1.53 a	38.60 ± 5.35 ab	89.70 ± 9.67 b	2.33 ± 0.07 a	0.08 ± 0.01 c
	M3	17.29 ± 1.86 ab	40.00 ± 2.28 ab	94.40 ± 5.61 ab	2.36 ± 0.05 a	0.13 ± 0.02 bc
	M4	17.53 ± 1.09 ab	38.60 ± 3.76 ab	86.80 ± 13.84 b	2.25 ± 0.29 a	0.16 ± 0.10 b
	HCK	16.42 ± 0.90 b	44.00 ± 1.90 a	96.80 ± 4.58 ab	2.20 ± 0.07 a	0.17 ± 0.03 a
	H1	19.88 ± 1.38 a	40.60 ± 1.85 b	91.40 ± 4.03 bc	2.25 ± 0.06 a	0.08 ± 0.02 b
	H2	18.70 ± 1.82 a	45.80 ± 1.72 a	100.60 ± 3.72 a	2.20 ± 0.11 a	0.12 ± 0.04 ab
	H3	18.98 ± 0.28 a	45.20 ± 0.98 a	98.00 ± 3.03 ab	2.17 ± 0.05 a	0.12 ± 0.04 ab
	H4	19.24 ± 1.71 a	44.00 ± 2.76 a	89.20 ± 6.97 c	2.03 ± 0.06 b	0.12 ± 0.04 ab
	FCK	16.42 ± 0.90 ab	43.80 ± 1.94 a	97.00 ± 4.60 a	2.21 ± 0.04 ab	0.17 ± 0.03 a
	F1	13.68 ± 1.58 c	33.80 ± 3.12 c	73.80 ± 7.33 d	2.18 ± 0.05 b	0.13 ± 0.02 b
	F2	15.38 ± 1.23 bc	37.60 ± 3.72 bc	83.60 ± 5.82 c	2.23 ± 0.06 ab	0.12 ± 0.00 b
	F3	17.64 ± 1.20 a	37.60 ± 3.26 bc	86.80 ± 6.91 bc	2.31 ± 0.04 a	0.11 ± 0.02 b
	F4	17.97 ± 1.85 a	41.40 ± 3.26 ab	94.00 ± 4.00 ab	2.28 ± 0.09 ab	0.14 ± 0.02 b

Note: Different letters in the table indicate significant difference at the 0.05 level among treatments on the same date (*p* < 0.05).

**Table 14:** Analysis of the interaction between spraying and sampling of different concentrations of MC on yield.

Variety	Index	Spraying Period		Treatment Concentration		Spraying Period × Treatment Concentration	
		F	Sig	F	Sig	F	Sig
HN65	Yield per Plant	45.58	<0.01	5.48	<0.01	4.31	<0.01
	Pods per Plant	35.75	<0.01	3.92	<0.01	4.39	<0.01
	Seeds per Plant	36.92	<0.01	3.16	0.02	4.53	<0.01
	Seeds per Pod	17.92	<0.01	1.83	0.13	2.53	0.02
	Empty grain rate	31.55	<0.01	5.33	<0.01	4.89	<0.01
HN44	Yield per Plant	17.28	<0.01	7.12	<0.01	4.89	<0.01
	Pods per Plant	16.1	<0.01	7.17	<0.01	1.61	0.14
	Seeds per Plant	11.29	<0.01	17.84	<0.01	4.68	<0.01
	Seeds per Pod	3.27	0.04	7.03	<0.01	8.24	<0.01
	Empty grain rate	4.55	0.01	5.17	<0.01	7.67	<0.01

**Table 15:** Basic physicochemical properties of the experimental soil used in the pot experiment.

Total Nitrogen (g/kg)	Total Phosphorus (g/kg)	Total Potassium (g/kg)	Alkali-Hydrolyzable Nitrogen (mg/kg)	Available Phosphorus (mg/kg)	Available Potassium (mg/kg)	Organic Matter (g/kg)	pH
0.79	1.01	18.31	126.67	39.59	180.59	31.53	7.21

**Table 16:** Test scheme.

Treatments	Spraying Time	Spraying MC Concentration (mg/L)	Frequency of Spraying (Times)
MCK	Seedling stage	0	2
M1	Seedling stage	50	2
M2	Seedling stage	100	2
M3	Seedling stage	200	2
M4	Seedling stage	400	2
HCK	Flowering stage	0	2
H1	Flowering stage	50	2
H2	Flowering stage	100	2
H3	Flowering stage	200	2
H4	Flowering stage	400	2
FCK	Seedling stage + Flowering stage	0	4
F1	Seedling stage + Flowering stage	50	4
F2	Seedling stage + Flowering stage	100	4
F3	Seedling stage + Flowering stage	200	4
F4	Seedling stage + Flowering stage	400	4

Note: For treatments with two sprays (M and H series), the second spray was applied 3 days after the first. For treatments with four sprays (F series), two sprays were applied at the seedling stage and two at the flowering stage, with a 3-day interval between each pair of sprays.

## Data Availability

Data will be made available on request.

## Abbreviations

The following abbreviations are used in this manuscript:

MC	mepiquat chloride
PGR	plant growth regulator
Pn	net photosynthetic rate
Gs	stomatal conductance
Tr	transpiration rate
Ci	intercellular CO_2_ concentration
Fv/Fm	maximum quantum efficiency of PSII
qL	photochemical quenching coefficient
NPQ	non-photochemical quenching
ΦII	effective quantum yield of PSII
ΦNPQ	quantum yield of regulated non-photochemical energy dissipation in PSII
ΦNO	quantum yield of non-regulated energy dissipation in PSII
PSII	photosystem II
